# Efficacy of Schisandra chinensis in liver injury: a systematic review and preclinical meta-analysis

**DOI:** 10.3389/fphar.2025.1627081

**Published:** 2025-08-04

**Authors:** Bo-Hao Huang, Bo-Han Lv, Dong-Jie Wu, Fei-Yang Xiong, Yan-Bo Li, Yan-Ping Lu, Wen-Liang Lv

**Affiliations:** ^1^ Graduate School, Beijing University of Chinese Medicine, Beijing, China; ^2^ Guang’an Men Hospital, China Academy of Chinese Medical Sciences, Beijing, China; ^3^ Shenzhen Bao’an Chinese Medicine Hospital, Guangzhou University of Chinese Medicine, Guangzhou, China

**Keywords:** Schisandra chinensis, liver injury, Chinese medicine, preclincal study, meta-analysis

## Abstract

**Background:**

Liver injury is a multifaceted condition marked by oxidative stress, inflammation, and apoptosis. Schisandra chinensis, a traditional Chinese medicinal herb with a history of use spanning over 2,000 years, exhibits significant hepatoprotective, antioxidant, anti-inflammatory, and anti-apoptotic effects. This study aims to review the therapeutic effects and underlying mechanisms of Schisandra chinensis in mitigating liver injury in animal models.

**Methods:**

A systematic review was conducted across eight databases. The methodological quality of the studies was assessed using the Sycle’s RoB tool. Sensitivity and subgroup analyses were performed in cases of high heterogeneity. Publication bias was evaluated using Egger’s test and funnel plots. A meta-analysis was carried out using Stata 18.0.

**Results:**

A total of 54 animal studies were included in this review. The results indicated that bioactive compounds in Schisandra chinensis significantly reduced levels of alanine aminotransferase (ALT) [standardized mean difference = −4.74, 95% confidence interval (−5.42, −4.06), p < 0.001, I^2^ = 90.8%], aspartate aminotransferase (AST) [SMD = −5.10, 95% CI (−5.84, −4.37), p < 0.001, I^2^ = 91.7%], and alkaline phosphatase (ALP). Additionally, Schisandra chinensis decreased malondialdehyde (MDA) levels while increasing superoxide dismutase (SOD) and glutathione (GSH). Additionally, the results revealed a significant reduction in pro-inflammatory cytokines, including Tumor Necrosis Factor-alpha (TNF-α), Interleukin-6 (IL-6), and Interleukin-1 beta (IL-1β). Subgroup analysis suggested that variations in animal species, drugs, modeling methods, and dosages may contribute to the observed heterogeneity.

**Conclusion:**

Schisandra chinensis demonstrates significant therapeutic effects in liver injury, likely due to its anti-inflammatory, antioxidant, and anti-apoptotic properties. However, further research is needed to validate its efficacy and safety.

**Systematic Review Registration:**

https://inplasy.com/inplasy-2025-2-0084/, identifier INPLASY202520084.

## Graphical Abstract

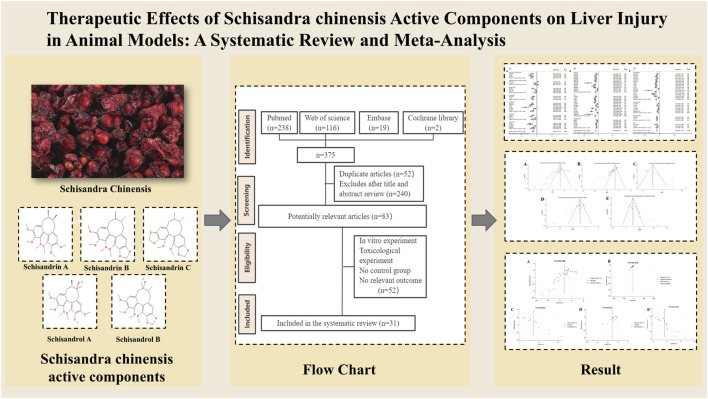

## 1 Introduction

Liver injury is characterized by hepatocellular damage, inflammatory reactions, reduced liver function, and the development of fibrosis (Tujios et al., 2022). The worldwide burden of liver injury has risen dramatically, making it the 11th leading cause of death globally (Devarbhavi et al., 2023). The etiology of liver injury is diverse, encompassing metabolic disorders, viral infections and toxin exposure, all of which trigger hepatocyte damage and fibrosis through oxidative stress, inflammatory cascades, and apoptosis (Taru et al., 2024). Despite significant advancements in the therapeutic management of hepatic injury, identifying safe and effective natural compounds for the prevention and treatment of hepatic injury remains crucial.

Schisandra chinensis (Turcz.) Baill., commonly known as Wu Wei Zi, is the dried mature fruit of the Schisandra genus. Its medicinal use dates back to the Eastern Han Dynasty (25-220 AD), as recorded in the Shennong Bencao Jing (Divine Farmer’s Materia Medica), where it was documented to have astringent, qi-tonifying, fluid-generating, kidney-nourishing, and heart-calming properties according to Traditional Chinese medicine (TCM) theories. Modern pharmacological studies have revealed that Schisandra chinensis exhibits anti-inflammatory, immunomodulatory, antitussive, and antiasthmatic properties (Yang et al., 2022), making it clinically valuable for treating disorders of the central nervous system (Li et al., 2023a), cardiovascular system (Shi et al., 2021), digestive system (Zhang et al., 2024), and endocrine system (Guo et al., 2025).

Meanwhile, Schisandra chinensis is a hepatoprotective herb, the renowned Ming Dynasty pharmacologist Li Shizhen explicitly noted in the Bencao Gangmu (Compendium of Materia Medica) that Schisandra chinensis used to treat liver deficiency syndromes. According to the Chinese Pharmacopoeia, the standard preparation consists of 3–9 g of dried berries, typically decocted in water for oral administration or processed into pills or powders. Research has revealed that the chemical composition of Schisandra chinensis fruit primarily consists of lignan compounds (e.g., Schisandrin A, Schisandrin B, Schisandrin C, Schisandrol A, and Schisandrol B), polysaccharides, and other bioactive constituents. These components are known to exhibit a range of pharmacological activities, including hepatoprotective, anti-inflammatory, antioxidant, and anti-apoptotic effects (Yang et al., 2022).

Many studies have demonstrated that Schisandra chinensis exhibits hepatoprotective effects in various murine models of liver injury. Furthermore, pharmaceutical agents derived from its Schisandra chinensis—such as bifendate (DDB) and bicyclol—have been extensively applied in clinical practice (Zhu et al., 2019). Extensive experimental studies have demonstrated that the bioactive ingredients of Schisandra chinensis can mitigate liver injury through multiple-target mechanisms, such as the nuclear factor erythroid-2-related factor 2 (Nrf2) signaling pathway (Zhao et al., 2022), nuclear factor-κB (NF-κB) signaling pathway (Yan et al., 2025), and NLRP3 inflammasome-mediated proptosis pathway (Bing et al., 2025).

While numerous studies have investigated the hepatoprotective effects of Schisandra chinensis, its precise molecular mechanisms and clinical translational potential remain unclear. A systematic review and meta-analysis are crucial for consolidating existing evidence and enhancing the reliability of these findings (Siddaway et al., 2019). Current evidence suggests no systematic evaluation exists regarding the therapeutic efficacy of Schisandra chinensis for hepatic injury. Therefore, this work attempts to systematically assess the protective effects and underlying mechanisms of Schisandra chinensis in models of liver injury.

## 2 Methods

The systematic review and meta-analysis were carried out in strict accordance with the PRISMA guidelines, ensuring a rigorous and transparent methodology (Page et al., 2021).

### 2.1 Search strategy

A comprehensive systematic search was performed across eight major databases: PubMed, Web of Science, Embase and Cochrane Library, CNKI, WANFANG, VIP, CBM, covering studies from their inception to January 2025. The search terms included “Acute Liver Injury,” “Chemically Induced Liver Toxicity,” and “Toxic Hepatitis,” as well as “Schisandra chinensis,” “schizandrol,” “schizandrin,” “schisantherin,” “gomisin,” and “Bay Starvine”. A detailed description of the search strategies employed for each database is provided in Supplementary Table S1 *.*

### 2.2 Inclusion and exclusion criteria

Following the PICO principle, the inclusion criteria were defined as follows: (1) participants: rats/mice with liver injury induced by drugs, alcohol, or chemical reagents; (2) intervention: extracts of Schisandra chinensis, lignans, Schizandrin, Schisandrin, Schizandrol, or other extracts from Schisandra chinensis with clearly defined administration time and dosages; (3) comparison: the control group received an equal volume of placebo solution (e.g., 0.9% saline, 1% carboxymethylcellulose, or vehicle-matched solvent); (4) outcomes: AST, ALT, ALP, SOD, GSH, and MDA levels; (5) papers: controlled *in vivo* experiments.

Studies meeting the following criteria were excluded: (1) non-original research literature, such as human clinical trials, case reports, meta-analyses, and systematic reviews; (2) non-holistic animal model studies, such as cell culture, isolated liver perfusion, or liver sectioning experiments; (3) conference abstracts, editorials, and letter articles not published in full text; (4) models that did not induce liver injury; (5) studies lacking relevant outcomes; (6) studies that did not have a concurrent control group (e.g., using only blank controls) or mismatched baseline data for the control group; (7) lack of sample sizes, margins of error, and statistical results (e.g., SD or SEM).

### 2.3 Data extraction

The retrieved literature was managed using NoteExpress (Version 9.0). After removing duplicates, two authors (Bo-Han Lv and Bo-Hao Huang) independently screened the studies and evaluated them according to the inclusion and exclusion criteria. To reach a consensus, any discrepancies were addressed by consulting the corresponding author (Wen-Liang Lv). Basic study information was recorded using Excel 2019, including:

1. The first author’s name and the publication year;
2. Type of animal model, sample size per group, body weight, and sex;
3. Liver injury modeling methods;
4. Drug type and dosage, with a particular focus on the highest administered dose, as well as the administration time and duration.
5. Inclusion of more than two active Schisandra ingredients as therapeutic agents in the study

If the study data were presented in graphical format, the authors were contacted to request the original numerical values. If the original data were unavailable, we used the digitization software GetData Graph Digitizer 2.26 to extract values from the graphs. When data were provided as standard error of the mean (SEM), we converted them into standard deviation (SD) using the formula SD = SEM × $\sqrt{n}$ , where n represents the number of animals per group.

### 2.4 Risk of bias assessment

Two investigators, Fei-Yang Xiong and Dong-Jie Wu, independently conducted a methodological quality assessment using SYRCLE’s Risk of Bias Tool (Hooijmans et al., 2014), which evaluates ten methodological aspects across various bias categories, including selection bias, performance bias, detection bias, attrition bias, reporting bias, and other biases. The evaluation system utilized three distinct symbols: “×” denoting minimal bias risk, “√” representing significant bias concerns, and “?” Signifying an indeterminate risk status due to inadequate data documentation. Discrepancies in assessment outcomes were resolved through consultation with a third evaluator, Bo-Hao Huang.

### 2.5 Statistical analysis

The statistical analyses were performed using Stata 18.0. The standardized mean difference (SMD) and 95% confidence interval (CI) were used to evaluate pooled outcome measures. Heterogeneity was assessed with the I^2^ statistic. A random-effects model was applied when I^2^ > 50% and *p* < 0.05; otherwise, a fixed-effects model was employed. Subgroup analyses were conducted based on variables such as animal species, model induction methods, treatment agents, and dosages to explore sources of heterogeneity. Sensitivity analyses were performed to assess the robustness of the results. Subgroup analyses for AST, ALT, SOD, MDA, and GSH were based on therapeutic drugs, mouse strains, modeling methods, and dosages. In studies with more than 10 studies, publication bias was assessed using funnel plots and Egger’s test. A symmetrical distribution of data points on the funnel plot indicated a low risk of publication bias, while asymmetry suggested potential bias. Egger’s test with p < 0.05 was considered indicative of significant publication bias.

## 3 Results

### 3.1 Study selection

**FIGURE 1:**
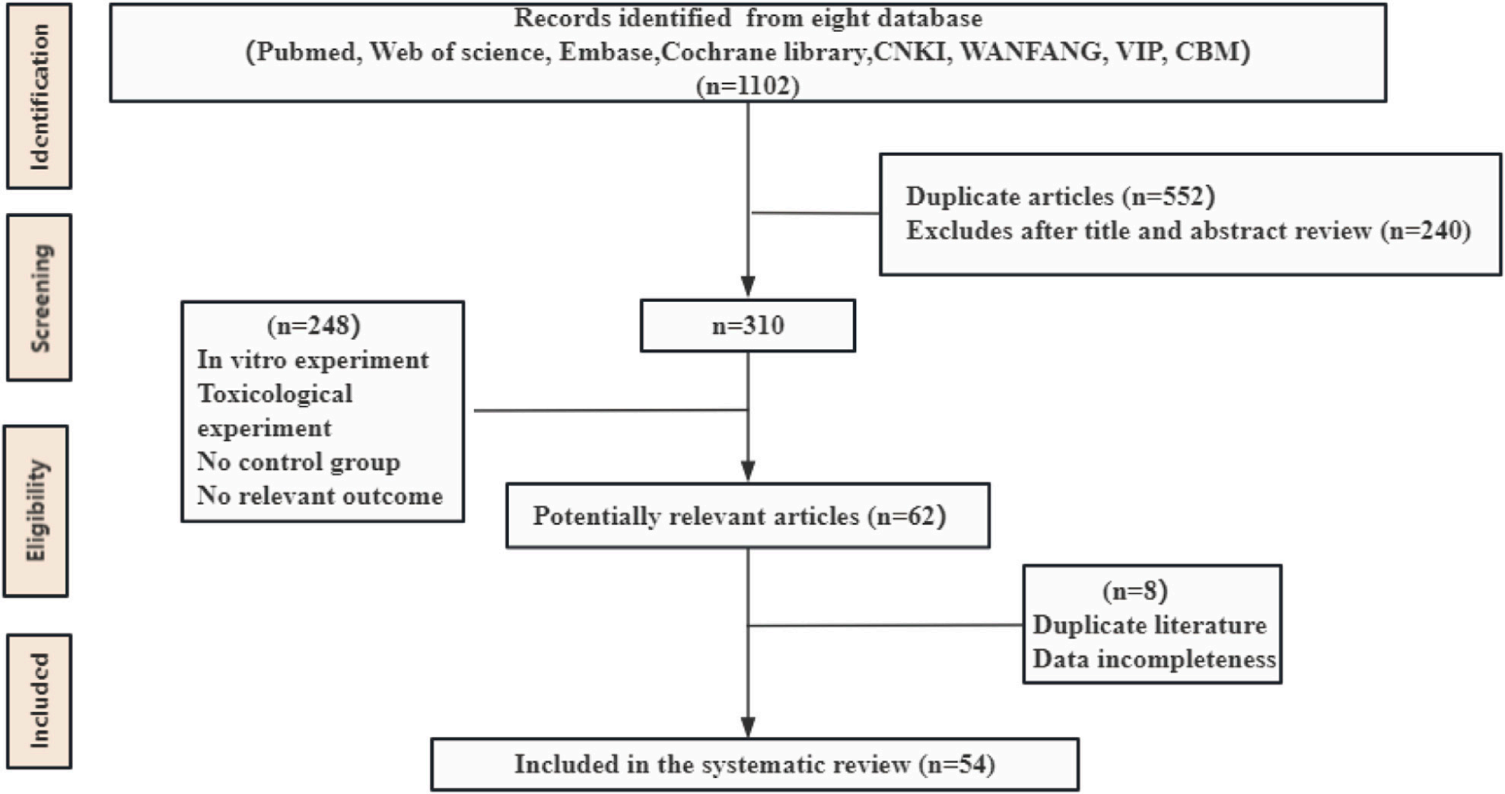
Flow diagram of database searches and study selection.

The initial database search identified 1,102 articles. After removing duplicates and reviewing articles, 792 records were excluded following a detailed screening of titles and abstracts. Subsequently, the remaining 310 articles underwent a full-text review, which was conducted independently by Bo-Hao Huang and Fei-Yang Xiong to ensure accuracy and consistency. After this rigorous assessment, 54 studies comprising 1,314 animals were selected for the final analysis. Details of the study selection process are depicted in Figure 1.

### 3.2 Study characteristics

Supplementary Table S2 provides an overview of the key features of the included studies, including: (1) first author, (2) publication year, (3) animal species, sample size per group, weight, and sex; (4) Model establishment method; (5) Interventional drug and dosage; (6) Mechanism of action; (7) Primary outcome measures.

Specifically, in this systematic review, 13 studies used C57BL/6 mice (Wang et al., 2014b; Jiang et al., 2015a, 2015b, 2016; Li et al., 2018; Nagappan et al., 2018; Zhai et al., 2018; Yan et al., 2021; Dai et al., 2022; Zhao et al., 2022; Chen et al., 2023; Lin et al., 2024, p. 38), six studies used Sprague-Dawley rats (Chen et al., 2017a; Ip et al., 1996; Shi et al., 2022; Su et al., 2019; Wei et al., 2021; Xie et al., 2014; Dai et al., 2022).

20 studies used ICR mice (Che et al., 2019a; Gao et al., 2016; Kim et al., 2008; Li et al., 2014a, 2020; Lu et al., 2014; Shan et al., 2019; Wang et al., 2014c; Yuan et al., 2018; Li et al., 2014b; Wang et al., 2014a; Li et al., 2013; Wang et al., 2020a; Guo et al., 2024; Che et al., 2019b; Wang et al., 2020b; Wang et al., 2019a, 2019b; Zhao et al., 2006), five study used Kunming rats (Chang et al., 2024; ZHOU et al., 2018; Yao et al., 2014; Sun, 2019; Yan et al., 2009), one study used BALB/c mice (Lam et al., 2023), and one study used Wistar rats (Teraoka et al., 2012).

The drugs used to establish liver injury animal models include: aflatoxin B (Ip et al., 1996), CdCl (Ip et al., 1996), Acetaminophen (Che et al., 2019a; Jiang et al., 2016, 2015a; 2015b; Li et al., 2014a, 2020; Wang et al., 2014b; Zhai et al., 2018; Zhao et al., 2022; Yan et al., 2009; Che et al., 2019b; Dai et al., 2022; Qiu et al., 2018), CCl4 (Teraoka et al., 2012; Xie et al., 2014; Chen et al., 2023), d-galactosamine (Gao et al., 2016; Li et al., 2013; Lu et al., 2014; Yan et al., 2021; Chen et al., 2014; Xu et al., 2011; ZHOU et al., 2018; Yao et al., 2014; Xu and Liu, 2011; Sun, 2019; Wang et al., 2020b, 2019a; Sun et al., 2021), ethanol solution (Lam et al., 2023; Li et al., 2018; Nagappan et al., 2018; Su et al., 2019; Wang et al., 2014c; Yuan et al., 2018; Xu et al., 2014; Li et al., 2014b; Wang et al., 2014a, 2019b), Concanavalin A (Shan et al., 2019; Li et al., 2013), Senecionine (Chang et al., 2024), Cyclophosphamide (Chen et al., 2017a), Cyclosporin A (Wei et al., 2021), LPS/D-GalN (Kim et al., 2008), lithocholic acid (Lin et al., 2024), dictamine (Chang et al., 2024), and pirarubicin (Shi et al., 2022).

The Schisandra chinensis extracts and active components used in the included studies were as follows: Schisandrae Chinensis Fructus extract (Chen et al., 2017a; Ip et al., 1996; Li et al., 2013, 2020; Wang et al., 2014b; Wei et al., 2021; ZHOU et al., 2018; Qiu et al., 2018; Yan et al., 2009; Zhao et al., 2006), Schisandra lignans (Su et al., 2019; Wang et al., 2014c; Xie et al., 2014; Xu et al., 2014; Yao et al., 2014; Wang et al., 2020a; An et al., 2014), Schisandrin A (Gao et al., 2016; Lu et al., 2014; Teraoka et al., 2012; Wang et al., 2020b, Wang et al., 2019a), Schisandrin B (Zhu and Wang, 2012; Li et al., 2014a, 2020; Jiang et al., 2015b; Gao et al., 2016; Sun et al., 2021; Shi et al., 2022; Lam et al., 2023), Schisandrin C (Chen et al., 2023; Jiang et al., 2015b; Dai et al., 2022), Schisandrol A (Jiang et al., 2015b; Li et al., 2020; Yan et al., 2021), Schisandrol B (Jiang et al., 2016; 2015a; 2015b; Li et al., 2020; Lin et al., 2024; Li et al., 2013), Schisantherin A (Li et al., 2018; Chang et al., 2024), Schisandra acid polysaccharides (Yuan et al., 2018; Che et al., 2019a), Schisandra polysaccharides (Shan et al., 2019; Xu et al., 2014; Li et al., 2014b; Chen et al., 2014; Wu et al., 2014; Wang et al., 2014a; Xu et al., 2011; Xu and Liu, 2011; Guo et al., 2024; Che et al., 2019b; Sun, 2019; Wang et al., 2019b), Schisandra essential oil (Zhai et al., 2018; Zhao et al., 2022), Gomisin A (Kim et al., 2008; Teraoka et al., 2012), and Gomisin N (Nagappan et al., 2018).

### 3.3 Evaluation of the methodological quality of selected studies

The quality of the included studies was independently evaluated by two researchers (Fei-Yang Xiong and Zi-Wen Zhuo) using SYRCLE’s Risk of Bias Tool (Hooijmans et al., 2014) across ten domains. The summarized risk of bias for each study is presented in the figure below. Forty-eight studies were assessed with a risk of bias score of 5, while four studies scored 6, and two studies scored 4.

**FIGURE 2:**
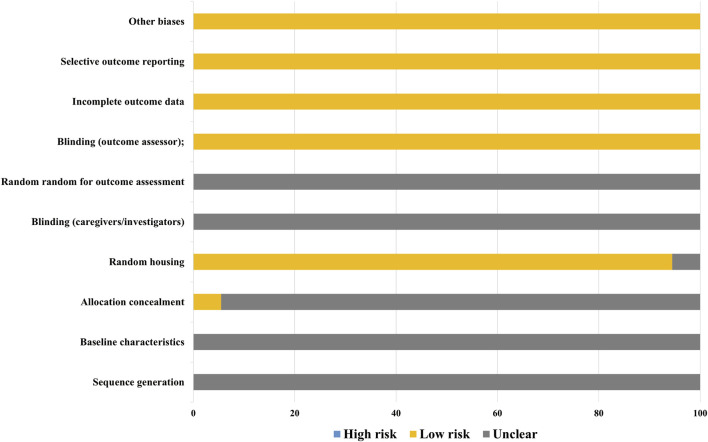
Risk of bias.

Among the 54 included studies, all adequately reported on animal inclusion and handled incomplete outcome data appropriately, ensuring comprehensive reporting of expected results. All randomized outcome-assessment studies were judged to be at low risk of bias. However, it should be noted that none of the included studies provided explicit descriptions regarding the implementation of allocation concealment or experimenter blinding procedures. Regarding other bias domains, all studies were evaluated as having low risk for incomplete outcome data, selective reporting, and other potential sources of bias (Figure 2; Supplementary Table S3).

### 3.4 Efficacy of Schisandra chinensis in treating liver injury

#### 3.4.1 Liver function

**FIGURE 3:**
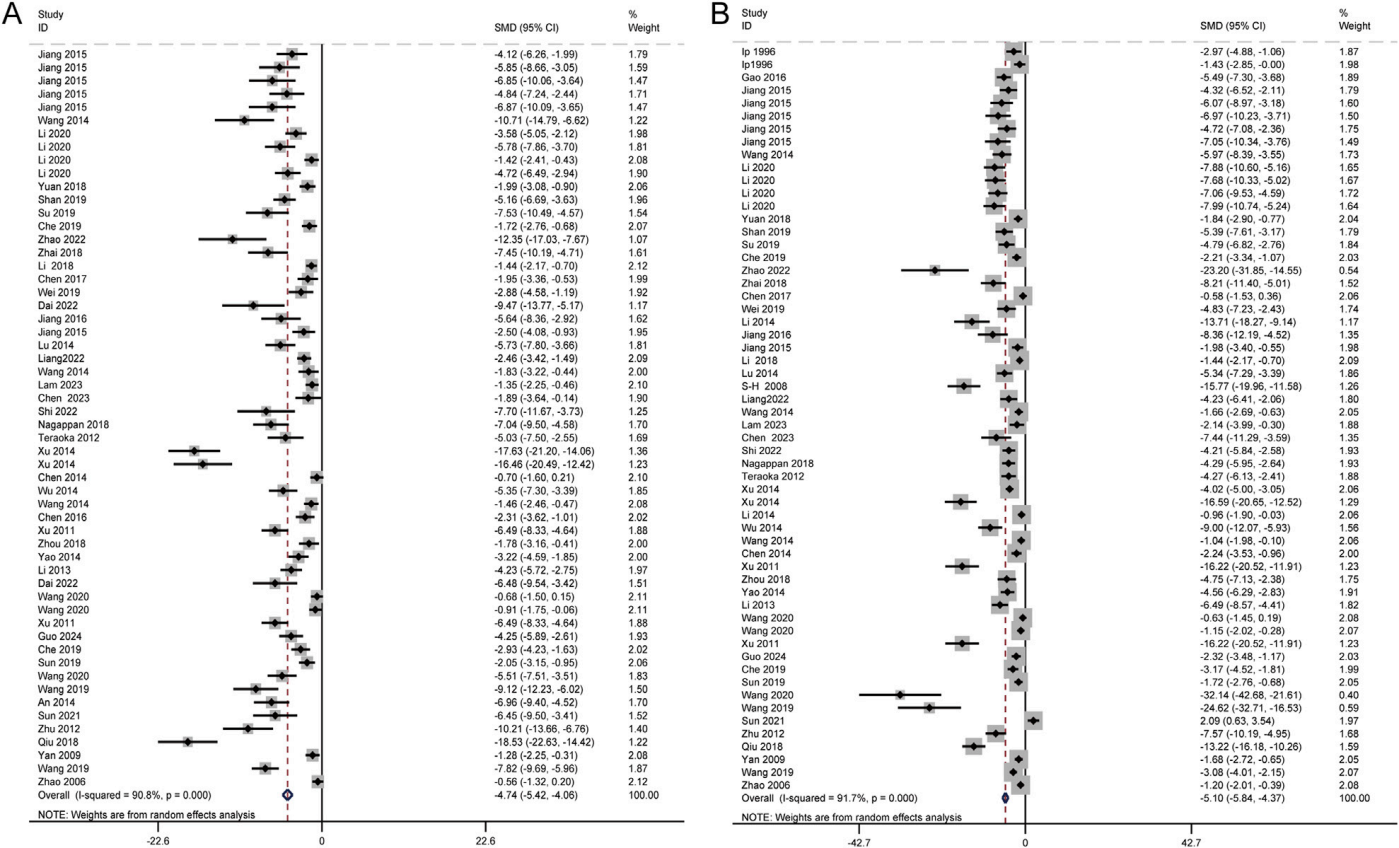
Forest plot (effect size and 95% CI) summarizing the effects of the Schisandra chinensis active ingredient on AST **(A)** and ALT **(B)**.

The levels of ALT and AST are widely recognized as the most reliable markers for detecting liver injury. A total of 46 studies, involving 1,150 animals, evaluated the effects of Schisandra chinensis on AST levels. According to the random-effects model, ALT levels were significantly lower in the experimental group, indicating Schisandra chinensis contributes to liver function improvement [SMD = −4.74, 95% CI (−5.42, −4.06), *p* < 0.001, I^2^ = 90.8%] (Figure 3A).

Similarly, 50 studies, including 1,184 animals, assessed the effects of Schisandra chinensis on ALT levels. The random-effects analysis revealed a significant reduction in ALT levels in the experimental group compared to the model group, suggesting a hepatoprotective effect of Schisandra chinensis [SMD = −5.10, 95% CI (−5.84, −4.37), *p* < 0.001, I^2^ = 91.7%] (Figure 3B).

**FIGURE 4:**
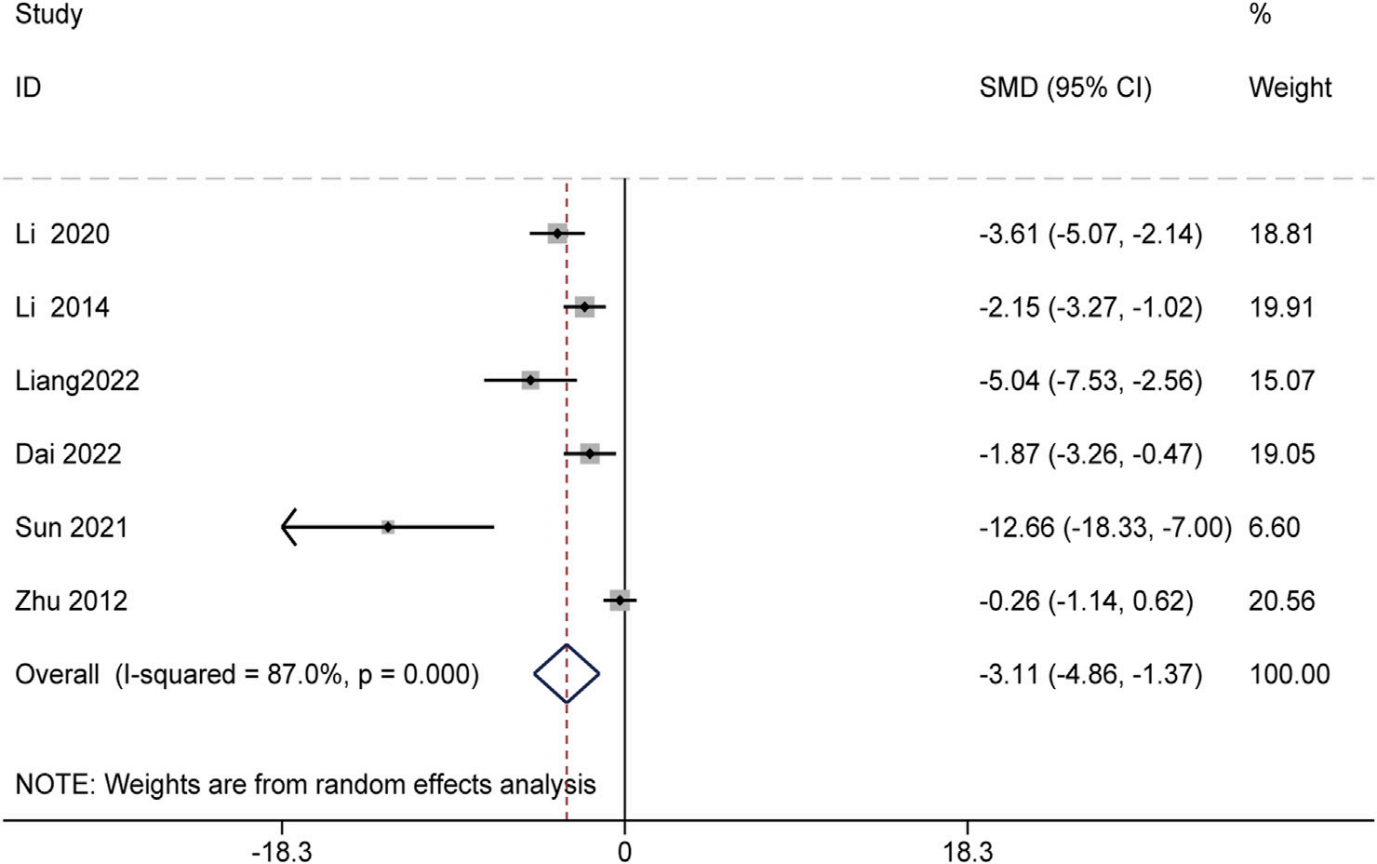
Forest plot (effect size and 95% CI) summarizing the effects of the Schisandra chinensis active ingredient on ALP.

Additionally, six studies involving 52 animals assessed the effects of Schisandra chinensis on ALP levels. A random-effects analysis suggested that, compared to the model group, the active components of Schisandra chinensis might reduce ALP levels [SMD = −3.11, 95% CI (−4.86, −1.37), *p* < 0.001, I^2^ = 87.0%] (Figure 4).

#### 3.4.2 Oxidative stress

**FIGURE 5:**
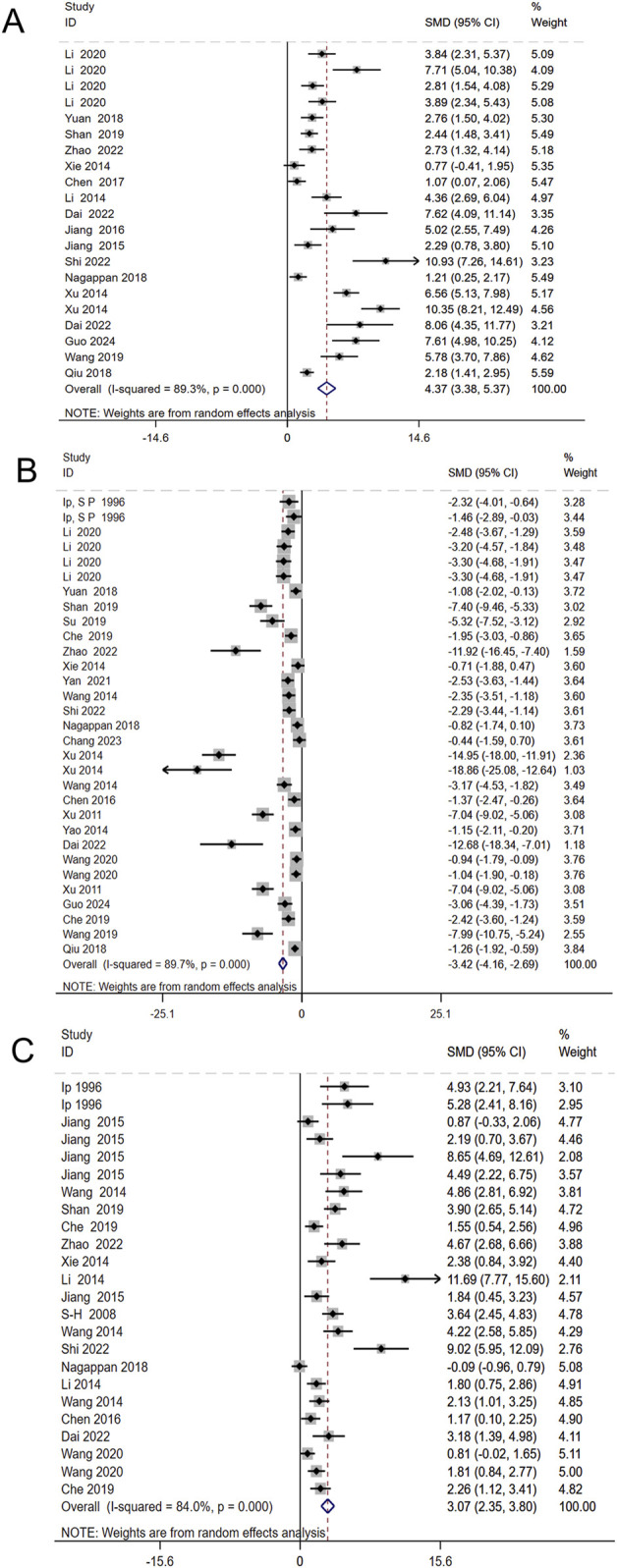
Forest plot (effect size and 95% CI) summarizing the effects of the Schisandra chinensis active ingredient on SOD **(A)**, MDA **(B)**, GSH **(C)**.

To investigate the regulatory effects of Schisandra chinensis on oxidative stress, 17 studies involving 466 animals assessed its impact on SOD levels. The results indicated that Schisandra chinensis significantly increased SOD levels [SMD = 4.37, 95% CI (3.38, 5.37), *p* < 0.001, I^2^ = 89.3%] (Figure 5A). Additionally, 26 studies involving 658 animals evaluated its effects on MDA levels, showing that Schisandra chinensis reduced MDA levels [SMD = −3.42, 95% CI (−4.16, −2.69), *p* < 0.001, I^2^ = 89.7%] (Figure 5B). Similarly, 20 studies involving 420 animals assessed its impact on GSH levels, revealing that Schisandra chinensis significantly increased GSH levels compared to the model group [SMD = 3.07, 95% CI (2.35, 3.80), *p* < 0.001, I^2^ = 84.0%] (Figure 5C).

#### 3.4.3 Inflammatory response

**FIGURE 6:**
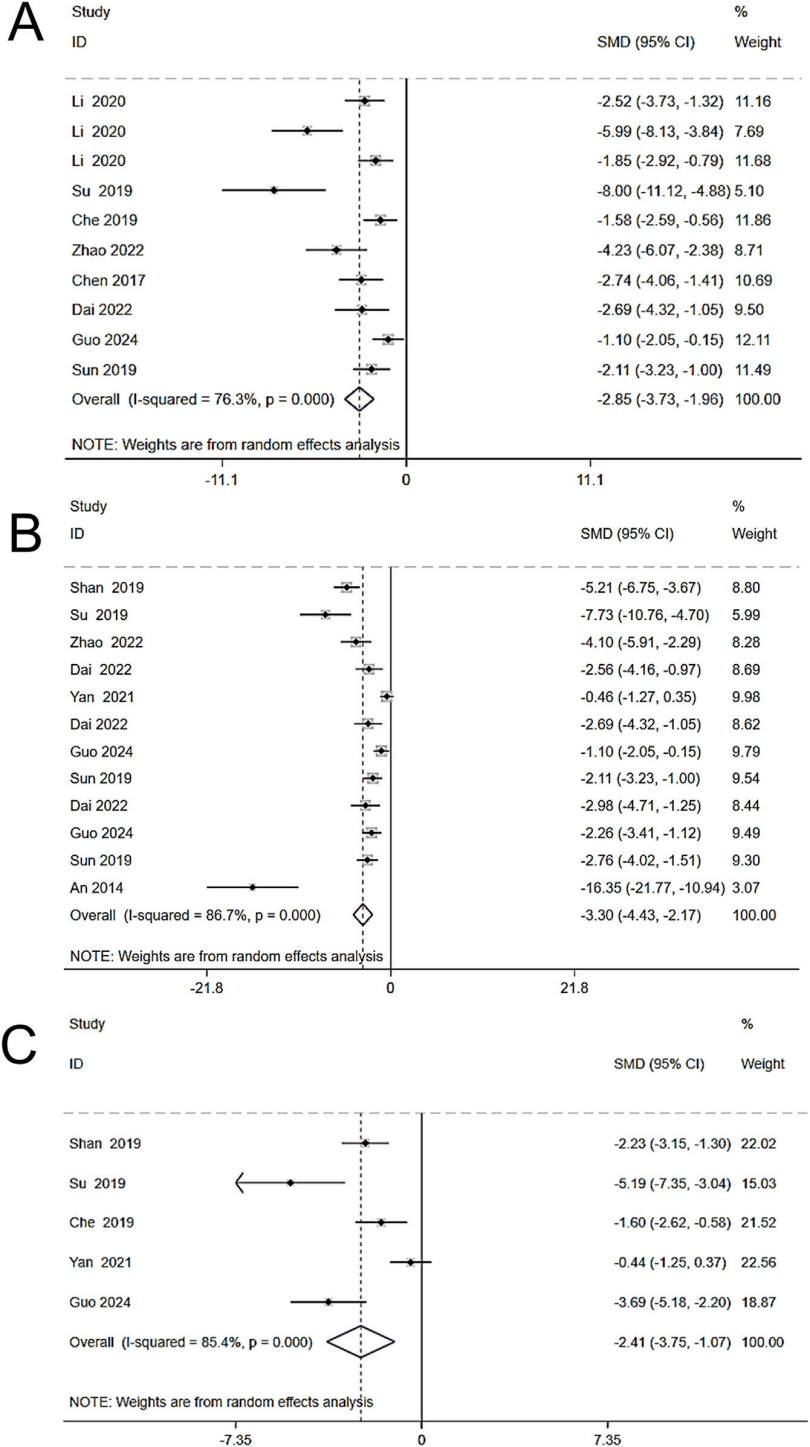
Forest plot (effect size and 95% CI) summarizing the effects of the Schisandra chinensis active ingredient on TNF-α **(A)**, IL-6 **(B)**, IL-1β **(C)**.

To investigate the effects of Schisandra chinensis on inflammation in liver injury, eight studies (including 182 animals) on TNF-α were included in a random effects analysis. The results showed that, compared to the model group, Schisandra chinensis significantly reduced serum TNF-α expression [SMD = −2.85, 95% CI (−3.73, −1.96), *p* < 0.001, I^2^ = 76.3%] (Figure 6A). 12 studies (including 222 animals) on IL-6 were included, and the results demonstrated that Schisandra chinensis significantly lowered IL-6 levels compared to the model group [SMD = −3.30, 95% CI (−4.43, −2.17), p < 0.001, I^2^ = 86.7%] (Figure 6B). Five studies (including 110 animals) showed that Schisandra chinensis reduced IL-1β levels [SMD = −2.41, 95% CI (−3.75, −1.07), *p* < 0.001, I^2^ = 85.4%] (Figure 6C).

#### 3.4.4 Lipid levels

**FIGURE 7:**
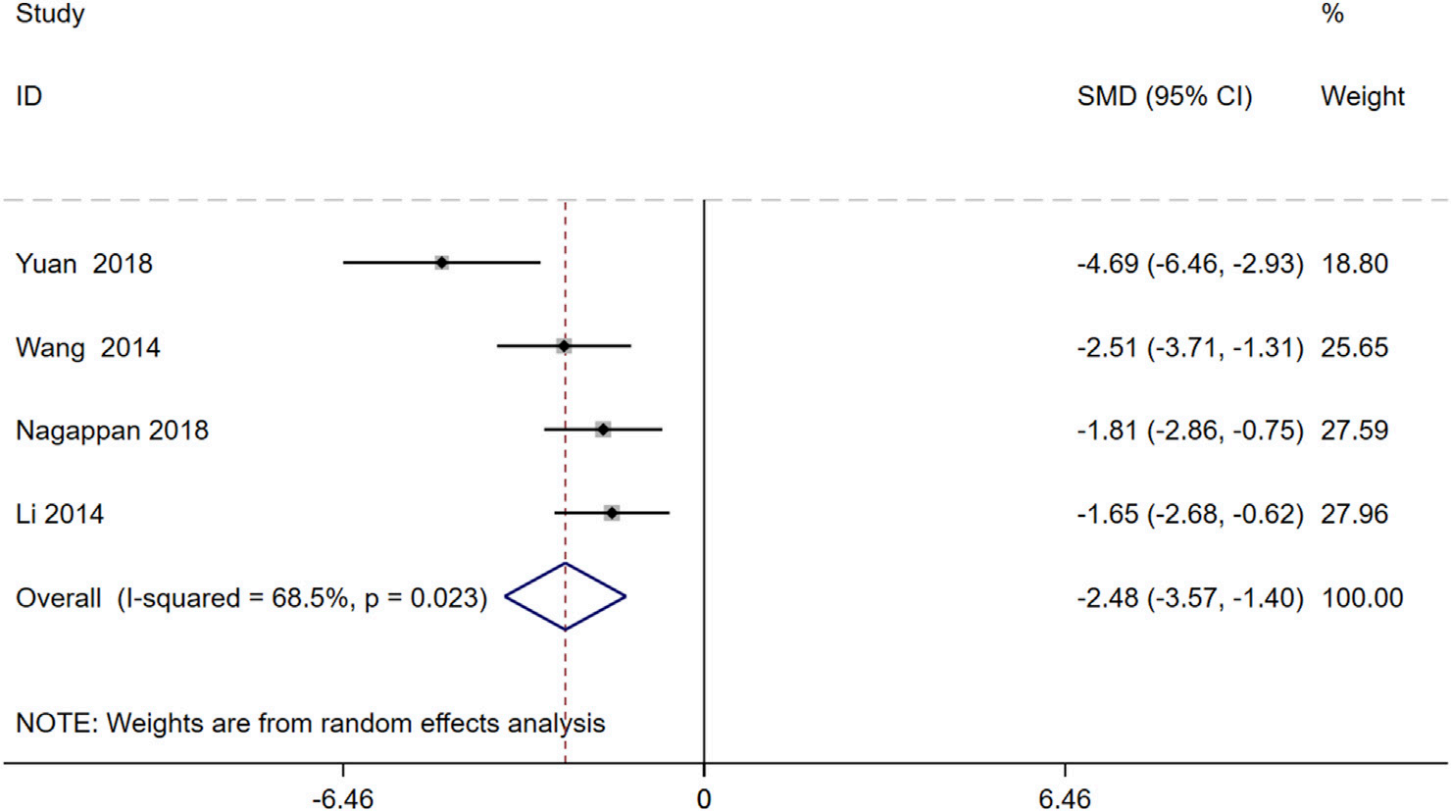
Forest plot (effect size and 95% CI) summarizing the effects of the Schisandra chinensis active ingredient on TG.

A random-effects analysis was conducted on four studies comprising 80 animals. The findings demonstrated that, compared to the model group, Schisandra chinensis significantly reduced triglyceride (TG) levels [SMD = −2.48, 95% CI -3.57, −1.40), *p* = 0.023, I^2^ = 68.5%] (Figure 7).

The effects of active components of Schisandra chinensis in treating liver injury are summarized in Table 1.

**TABLE 1:** Summary of the effects of active components of Schisandra chinensis on liver injury.

Indicator	Outcomes	Number of studies	Sample size	SMD (95% CI)	I^2^ (%)	*P*
Liver function	AST	46	1,150	−4.74, (−5.42, −4.06)	90.8	<0.001
	ALT	50	1,184	−5.10, (−5.84, −4.37)	91.7	<0.001
	ALP	6	52	3.11, (−4.86, −1.37)	87.0	<0.001
Oxidative Stress	SOD	17	466	4.37, (3.38, 5.37)	89.3	<0.001
	MDA	26	658	−3.42, (−4.16, −2.69	89.7	<0.001
	GSH	20	420	3.11, (2.35, 3.80)	87.0	<0.001
Lipid	TG	6	52	−3.11, (−4.86, −1.37)	87.0	<0.001
Inflammatory Response	TNF-α	8	182	−2.85, (−3.73, −1.96	76.3	<0.001
	IL-6	12	222	−3.30, (−4.43, −2.17)	86.7	<0.001
	IL-1β	5	110	−2.41, (−3.75, −1.07)	85.4	<0.001

### 3.5 Subgroup analysis

To address the considerable heterogeneity observed across studies, we performed subgroup analyses focusing on three confounding factors (animal species, treatment agents, model drugs and dosages), to identify the sources of heterogeneity for the five primary outcomes (AST, ALT, SOD, MDA, GSH).

#### 3.5.1 Subgroup analysis of AST levels

Compared with the model group, all intervention groups demonstrated significant reductions in AST levels. Notably, in the treatment agent subgroup, Schisandrin C showed the most significant effect [SMD = −5.77, 95% CI (−10.45, −1.09), *p* = 0.001, I^2^ = 86.4%].In the animal subgroup, Sprague-Dawley rats exhibited the most pronounced effect [SMD = −7.68, 95% CI (−11.12, −4.25), *p* < 0.001, I^2^ = 92.6%]. In the model drug subgroup, the ethanol solution group showed the most significant improvement [SMD = −6.09, 95% CI (−8.51, −3.55), *p* < 0.001, I^2^ = 95.9%]. Among dosages, the high-dose group did not differ significantly from the low-dose group, and the effect size was even greater in the low-dose group [SMD = −5.00, 95% CI (−6.04, −3.95), *p* < 0.001, I^2^ = 90.6%]. However, the heterogeneity remained unresolved after the subgroup analysis, suggesting that these three factors may be significant sources of heterogeneity (Table 2; Supplementary Figure S1).

**TABLE 2:** Subgroup analysis of AST levels.

Indicator	Subgroup	Number of studies	SMD (95% CI)	I^2^ (%)	*P*
Therapeutic drug	Schisandra Chinensis Extract	7	−4.69 (−6.92, −2.45)	94.0	<0.001
	Schisandra Polysaccharides	13	−4.90 (−6.46, −3.33)	93.6	<0.001
	Schisandra Acidic Polysaccharides	2	−1.85 (−2.60, −1.10)	0.0	0.725
	Lignans Extract	8	−4.52 (−6.57,-2.47)	93.9	<0.001
	Schisandrin A	3	−5.78 (-7.49,-4.07)	70.4	0.034
	Schisandrin B	4	−4.15 (−6.49,-1.82)	78.3	0.003
	Schisandrin C	3	−5.77 (−10.45,-1.09)	86.4	0.001
	Schisandrol A	2	−2.95 (−6.82,0.38)	84.9	0.010
	Schisandrol B	3	−4.09 (−5.95, −2.23)	62.8	0.068
Species	C57BL/6 mice	12	−5.10 (−6.38, −3.82)	85.5	<0.001
	Kunming mice	4	−1.85 (−2.42, −1.28)	0.0	0.450
	Sprague-Dawley rats	7	−7.68 (−11.12,-4.25)	92.6	<0.001
	ICR mice	16	−3.34 (−4.11,-2.57)	87.9	<0.001
Modeling drugs	Acetaminophen	8	−5.87 (−7.40,-4.33)	90.6	<0.001
	Ethanol solution	8	−6.03 (−8.51,-3.55)	95.9	<0.001
	CCL4	7	−4.22 (-3.71,-2.61)	86.9	<0.001
Dosages	≥100 mg kg^−1^	27	−4.62 (−5.59, −3.65)	89.3	<0.001
	<100 mg kg^−1^	24	−4.70 (−5.42, −3.97)	90.6	<0.001

#### 3.5.2 Subgroup analysis of ALT levels

Compared with the model group, all intervention groups demonstrated significant reductions in ALT levels. Specifically, among treatment agent, Schisandrin C demonstrated the largest effect size with concomitant reduction in heterogeneity [SMD = −7.52, 95% CI (−10.13, −4.91), I^2^ = 0.0%, *p* = 0.579]. Among animal species, Sprague-Dawley rats showed the most pronounced effect [SMD = −4.26, 95% CI (−4.99, −3.52), *p* < 0.001, I^2^ = 89.8%]. Among model drugs, acetaminophen showed the most significant improvement [SMD = −5.08, 95% CI (−5.60, −4.57)*, p* < 0.001]. Among dosages, the effect was most marked at elevated doses [SMD = −5.00, 95% CI (−6.04, −3.95), *p* < 0.001, I^2^ = 90.6%]. The unresolved heterogeneity after subgroup stratification strongly suggests that these three variables are primary contributors to between-study differences (Table 3; Supplementary Figure S2).

**TABLE 3:** Subgroup analysis of ALT levels.

Indicator	Subgroup	Number of studies	SMD (95% CI)	I^2^ (%)	*P*
Therapeutic drugs	Schisandra Chinensis Extract	7	−5.57 (−8.12, −3.01)	93.7	<0.001
	Schisandra Polysaccharides	12	−4.27 (−5.62, −2.91)	92.1	<0.001
	Schisandra Acidic Polysaccharides	2	−2.01 (−2.79, −1.23)	0.00	0.638
	Lignans Extract	6	−2.56 (−4.04, −1.08)	90.4	0.001
	Schisandrin A	2	−4.89 (−6.35, −3.43)	0.00	0.498
	Schisandrin B	6	−6.00 (−8.25, −3.76)	82.7	<0.001
	Schisandrin C	3	−7.52 (−10.13, −4.91)	0.0	0.579
	Schisandrol A	3	−4.32 (−7.75, −0.89)	89.2	<0.001
	Schisandrol B	4	−5.36 (−8.46, −2.27)	85.5	<0.001
Modeling drugs	Acetaminophen	14	−5.08 (−5.60, −4.57)	86.6	<0.001
	Ethanol solution	5	−2.01 (−2.48, −1.55)	71.3	0.004
	CCL4	10	−3.66 (−4.20, −3.11)	90.0	<0.001
Species	C57BL/6 mice	9	−4.15 (−4.79, −3.52)	85.0	<0.001
	ICR mice	21	−2.41 (−2.66, −2.15)	90.2	<0.001
	Sprague-Dawley rats	6	−4.26 (−4.99, −3.52)	89.8	<0.001
	Kunming mice	4	−2.34 (−2.99, −1.69)	77.3	0.004
Dosages	≥100 mg kg^−1^	26	−5.00 (−6.04, −3.95)	90.6	<0.001
	<100 mg kg^−1^	21	−4.18 (−5.20, −3.16)	89.6	<0.001

#### 3.5.3 Subgroup analysis of SOD levels

Compared with the model group, all intervention groups demonstrated a significant advance in SOD levels. Specifically, among treatment agents, Schisandrin B showed the most significant effect size [SMD = 5.31, 95% CI (3.89,6.73), I^2^ = 77.0%, *p* = 0.037]. Among animal species, ICR mice demonstrated the largest effect size, accompanied by a concomitant reduction in heterogeneity [SMD = 3.27, 95% CI (2.75,3.80), *p* = 0.008, I^2^ = 65.5%]. Among dosages, higher dosage levels exhibited the most significant impact [SMD = 5.18, 95% CI (3.12,7.25)), *p* < 0.001, I^2^ = 92.8%]. However, heterogeneity has improved to a lesser extent (Table 4; Supplementary Figure S3).

**TABLE 4:** Subgroup analysis of SOD levels.

Indicator	Subgroup	Number of studies	SMD (95% CI)	I^2^ (%)	*P*
Therapeutic drugs	Schisandra Chinensis Extract	3	2.05 (1.48,2.61)	78.1	0.010
	Schisandrin B	2	5.31 (3.89,6.73)	77.0	0.037
	Schisandrol B	3	3.38 (2.39,4.37)	51.2	0.129
Species	C57BL/6 mice	4	3.22 (2.30,4.13)	69.8	0.019
	ICR mice	4	3.27 (2.75,3.80)	65.5	0.008
	Sprague-Dawley rats	7	2.54 (1.91,3.16)	92.5	<0.001
Dosages	≥100 mg kg^−1^	10	5.18 (3.12,7.25)	92.8	<0.001
	<100 mg kg^−1^	7	3.25 (2.06, 4.44)	84.0	<0.001

#### 3.5.4 Subgroup analysis of MDA levels

All treatment groups exhibited statistically significant improvements in MDA levels relative to the control group. Specifically, among treatment agents, Schisandrol A demonstrated the largest effect size, accompanied by a concomitant reduction in heterogeneity [SMD = −2.88, 95% CI (−3.41, −2.34), I^2^ = 69.3%, *p* = 0.003]. Among model drugs, acetaminophen showed the most significant improvement [SMD = −5.08, 95% CI (−5.60, −4.57), *p* < 0.001]. Among animal species, C57BL/6 mice showed the most pronounced effect [SMD = −6.96, 95% CI (−16.15, 2.23), *p* < 0.001, I^2^ = 93.6%]. Among dosages, higher doses demonstrated the most pronounced effect [SMD = −4.70, 95% CI (−6.30,-3.11), *p* < 0.001, I^2^ = 92.8%]. However, heterogeneity has improved less (Table 5; Supplementary Figure S4).

**TABLE 5:** Subgroup analysis of MDA levels.

Indicator	Subgroup	Number of studies	SMD (95% CI)	I^2^ (%)	*P*
Therapeutic drugs	Schisandra Chinensis Extract	3	−1.61 (−2.12, −1.09)	22.4	0.296
	Schisandra Polysaccharides	8	−2.60 (−3.07, −2.13)	91.6	<0.001
	Lignans Extract	3	−2.00 (−2.78, −1.23)	87.4	<0.001
	Schisandrol A	2	−2.82 (−3.68, −1.96)	0.00	0.396
Modeling drugs	Acetaminophen	4	−2.61 (−3.10, −2.11)	0.0	0.481
	Ethanol solution	4	−2.21 (−2.83, −1.59)	80.3	0.002
	D-galactosamine	3	−2.15 (−2.88, 1.42)	86.8	0.001
Species	C57BL/6 mice	2	−6.96 (−16.15,2.23)	93.6	<0.001
	Sprague-Dawley rats	6	−2.31 (−3.39, −1.22)	67.3	0.009
	ICR mice	15	−3.00 (−3.78, −2.22)	84.9	<0.001
Dosages	≥100 mg kg^−1^	14	−4.70 (−6.30, −3.11)	92.8	<0.001
	<100 mg kg^−1^	13	−2.68 (−3.57, −1.80)	87.4	<0.001

#### 3.5.5 Subgroup analysis of GSH levels

All treatment groups exhibited statistically significant improvements in GSH levels relative to the control group. Specifically, among treatment agents, Schisandra chinensis extract demonstrated the largest effect size, accompanied by a concomitant reduction in heterogeneity [SMD = −4.99, 95% CI (3.56, 6.41), I^2^ = 0.0%, *p* = 0.927]. Among animal species, C57BL/6 mice showed the most pronounced effect [SMD = 2.87, 95% CI (2.19, 3.54), *p* < 0.001, I^2^ = 79.1%]. Among dosages, higher doses demonstrated the most pronounced effect [SMD = 3.49, 95% CI (2.40, 4.58), *p* < 0.001, I^2^ = 80.0%]. Notably, the heterogeneity persisted despite intervention (Table 6; Supplementary Figure S5).

**TABLE 6:** Subgroup analysis of GSH levels.

Indicator	Subgroup	Number of studies	SMD (95% CI)	I^2^ (%)	*P*
Therapeutic drugs	Schisandra chinensis polysaccharide	6	1.57 (1.16, 1.98)	22.8	0.263
	Schisandra Chinensis Extract	2	4.99 (3.56, 6.41)	0.0	0.927
	Lignans Extract	4	1.80 (1.22, 2.37)	79.6	0.002
Species	C57BL/6 mice	4	2.82 (2.18, 3.47)	72.3	<0.001
	ICR mice	3	1.13 (0.57, 1.68)	0.0	0.544
	Wistar rats	2	2.47 (1.62, 3.32)	95.3	<0.001
Dosages	≥100 mg kg^−1^	13	3.49 (2.40, 4.58)	80.0	<0.001
	<100 mg kg^−1^	10	2.84 (1.74, 3.95)	88.3	<0.001

### 3.6 Sensitivity analysis

Sensitivity analysis was conducted by sequentially excluding individual studies and re-running the combined analysis for AST, ALT, SOD, MDA, and GSH. The results showed no significant changes after excluding certain studies, indicating that the findings are robust and reliable (Supplementary Tables S4–S8).

### 3.7 Publication bias analysis

**FIGURE 8:**
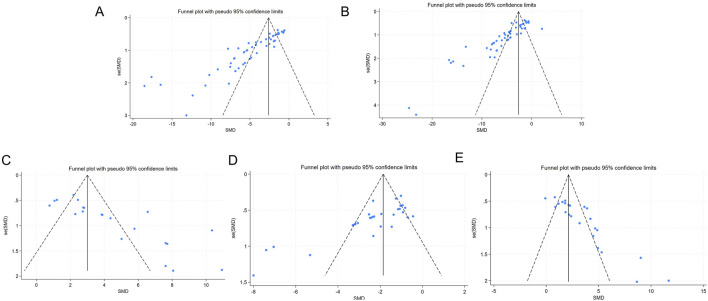
Funnel chart **(A)** AST; **(B)** ALT **(C)** SOD; **(D)** MDA; **(E)** GSH.

**FIGURE 9:**
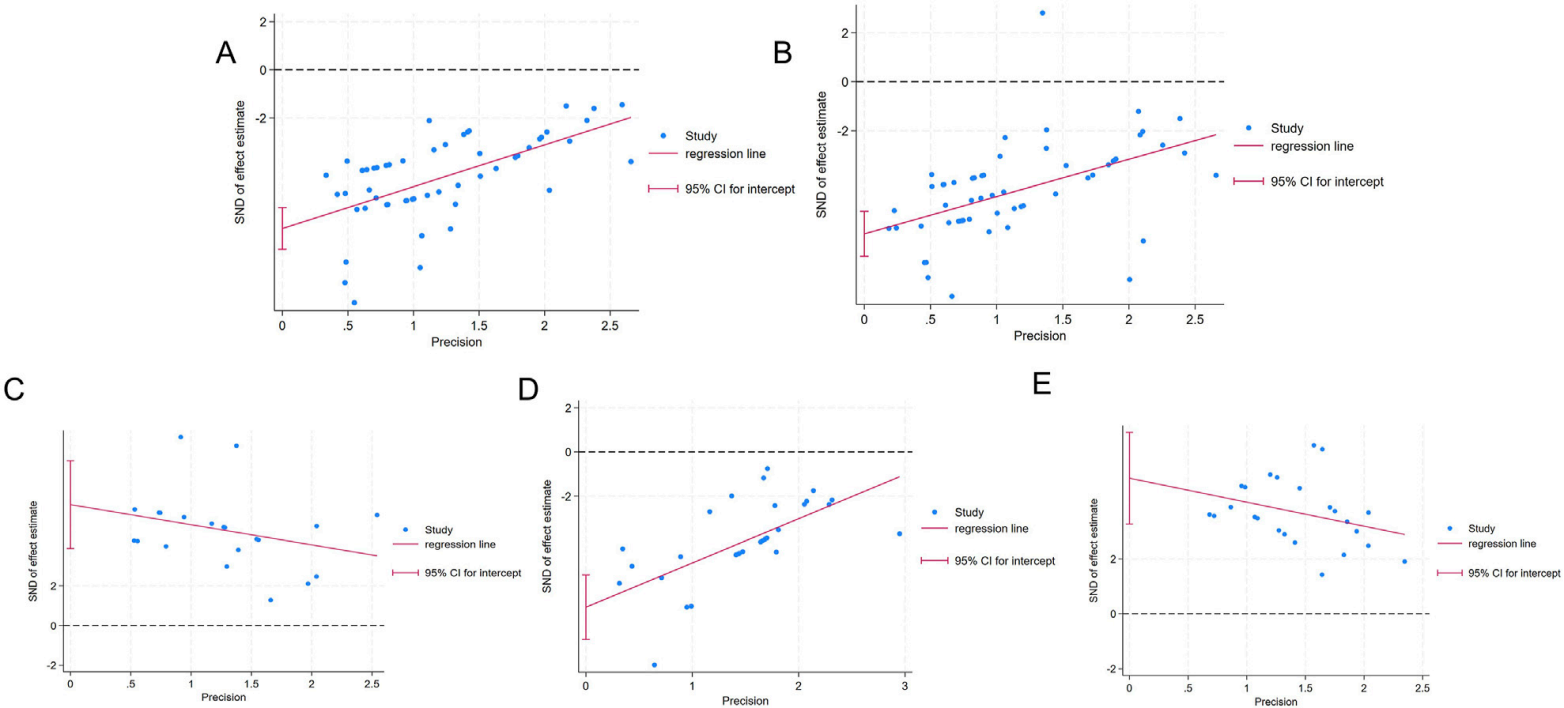
Egger’s publication bias **(A)** AST **(B)** ALT; **(C)** SOD **(D)** MDA; **(E)** GSH.

Funnel plots and Egger’s test were utilized to evaluate publication bias for the five outcomes related to liver function and oxidative stress. The funnel plot showed asymmetry between studies on both sides, suggesting the potential presence of publication bias (Figure 8). The results of Egger’s test indicated statistically significant publication bias for AST (*p* < 0.05), ALT (*p* < 0.05), SOD (*p* < 0.05), MDA (*p* < 0.05), and GSH (*p* < 0.05) (Figure 9).

The trim-and-fill method was then employed to identify potential missing studies and evaluate asymmetry. The results indicated that the data from the missing studies would not alter the magnitude of the overall summary effect (Table 7; Supplementary Figure S6).

**TABLE 7:** Egger’s test and trim-and-fill analysis.

Parameter	Before trim and fill			After trim and fill		
	*P* value	SMD	No. studies	*P* value	SMD	No. studies
AST	*p* < 0.05	−5.044	55	*p* < 0.05	−5.611	61
ALT	*p* < 0.05	−2.705	59	*p* < 0.05	−2.705	59
SOD	*p* < 0.05	4.521	21	*p* < 0.05	4.521	21
MDA	*p* < 0.05	−3.970	31	*p* < 0.05	−3.970	31
GSH	*p* < 0.05	2.647	24	*p* < 0.05	2.647	24

## 4 Discussion

### 4.1 Summary of evidence

This systematic review and meta-analysis indicate that the active components of Schisandra chinensis exhibit significant protective effects against liver injury in animal models. The findings demonstrate that Schisandra chinensis possesses remarkable hepatoprotective properties, as evidenced by its ability to significantly reduce levels of ALT, AST, and ALP, which are widely accepted biomarkers of liver injury.

In addition to its effects on liver function markers, Schisandra chinensis has been shown to enhance SOD activity and increase GSH levels, both of which are crucial in reducing oxidative stress. Simultaneously, it substantially lowered MDA levels, a significant indicator of oxidative damage and lipid peroxidation. Furthermore, Schisandra chinensis has demonstrated anti-inflammatory effects, as evidenced by its ability to reduce levels of significant inflammatory markers, including TNF-α, IL-6, and IL-1β.

While these studies show positive outcomes, considerable variation remains among the key measurements. Subgroup analyses were conducted to identify potential sources of this variation, revealing that factors such as animal species, treatment strategies, modeling approaches, and dosages may account for these differences. Egger’s test was performed to assess potential publication bias for AST, ALT, SOD, GSH, and MDA. The results showed no significant publication bias, thus enhancing the reliability and robustness of the conclusions.

### 4.2 Hepatoprotective effects and molecular mechanisms of Schisandra chinensis

#### 4.2.1 Anti-inflammatory properties

Inflammation, primarily caused by the excessive synthesis of inflammatory cytokines such as TNF-α, IL-6, and IL-1β, plays a central role in liver damage. (Malhi and Gores, 2008). Recent studies have highlighted the anti-inflammatory mechanisms of Schisandra chinensis. For instance, its bioactive component Schizandrin C has been shown to inhibit the phosphorylation of p38 MAP kinase and extracellular signal-regulated protein kinase (ERK), thereby lowering the levels of TNF-α, IL-6, and IL-1β, which contribute to its hepatoprotective effects (Chen et al., 2023). Additionally, the Schisandra acidic polysaccharide has been shown to activate the Adenosine 5′-monophosphate-activated protein kinase (AMPK) and Protein kinase B (Akt) signaling pathways, leading to a reduction in TNF-α and IL-1β levels (Che et al., 2019a). Li et al. observed that Schisandra chinensis downregulates NF-κB expression in acute liver injury models (Li et al., 2018).

Network pharmacology studies by Xu et al. (2021) revealed that Schisandrol B suppresses pro-inflammatory cytokine expression by downregulating Inducible nitric oxide synthase (iNOS) and Cyclooxygenase-2 (COX-2) through the Interleukin-17 (IL-17) signaling pathway. *In vitro* studies have also demonstrated that Schisandrin B exerts anti-inflammatory effects by modulating redox-sensitive transcription factors, such as Nrf2 and NF-κB (Checker et al., 2012). Moreover, Schisandra chinensis also regulates the Toll-like receptor 4 (TLR4)/Myeloid differentiation primary response 88 (MyD88) signaling pathway to prevent inflammasome activation, thereby reducing the release of IL-1β and IL-18, and mitigating hepatocyte inflammation. These findings underscore the multiple anti-inflammatory actions of Schisandra chinensis, which contribute to its hepatoprotective properties.

#### 4.2.2 Antioxidant effects

Reactive oxygen species (ROS), when excessively accumulated in hepatocytes, cause oxidative stress, leading to DNA damage, protein oxidation, and lipid peroxidation (Zheng et al., 2019). Mitigating oxidative stress-induced liver injury is crucial, not only through direct ROS scavenging but also via activation of Nrf2, which regulates downstream genes associated with antioxidant defense (Shen et al., 2017).

Schisandra chinensis polysaccharides have been shown to upregulate Nrf2 and heme oxygenase-1 (HO-1) while significantly lowering the expression of Kelch-like ECH-associated protein 1 (Keap1) (Shan et al., 2019). Concurrently, Schisandra chinensis polysaccharides downregulate TLR4 and NF-κB expression, hence lowering oxidative stress. In the CCl4-induced liver injury model, Schisandrin B was shown to activate the Nrf2/Antioxidant Response Element (ARE) signaling pathway, upregulate the expression of antioxidant genes, and enhance cellular antioxidant capacity, ultimately reducing hepatocyte damage (Chen et al., 2017b). By lowering TNF-α, IL-1β, and IL-6 levels and thereby suppressing Cytochrome P450 proteins (CYP2E1), lignan extracts from Schisandra chinensis have also demonstrated effectiveness in reducing alcohol-induced hepatic inflammation (Yuan et al., 2018). Additionally, they enhance the activation of Nrf2, HO-1, Glutamate-cysteine Ligase (GCLM), and NAD(P)H quinone dehydrogenase 1 (NQO1), further reinforcing their antioxidant properties (Su et al., 2019). By improving detoxification and increasing antioxidant capacity, Schisandrol B exhibits notable protective effects against acetaminophen-induced hepatotoxicity through the activation of the Nrf2/ARE pathway (Jiang et al., 2015a). These results collectively suggest that Schisandra chinensis possesses hepatoprotective properties through multiple antioxidant systems, underscoring its potential therapeutic value in the treatment of liver damage.

#### 4.2.3 Anti-apoptotic effects

Schisandra chinensis demonstrates hepatoprotective properties through its anti-apoptotic action. Studies have shown that acidic polysaccharides from Schisandra chinensis reduce the BCL2-associated X protein (Bax)/B-cell lymphoma 2 (Bcl-2) ratio, inhibit caspase-3 expression, and upregulate p-AMPK, p-Akt, and phospho-Glycogen Synthase Kinase 3 beta (p-GSK3β) expression in acetaminophen-induced liver injury models (Che et al., 2019a). Moreover, Schisandrol B reduces atypical cell death induced by Apoptotic protease-activating factor 1 (Apaf-1) inflammasomes through the PXR/Forkhead box protein O1 (FoxO1)/Apaf-1 axis, thereby mitigating cholestasis-induced liver damage (Liang et al., 2022). By increasing the expression of Cyclin D1 (CCND1), Proliferating Cell Nuclear Antigen (PCNA), and BCL-2, Schisandrol B also reduces acetaminophen-induced activation of p53 and p21, thereby promoting liver regeneration (Jiang et al., 2015a). Lignan compounds, such as gomisin A, protect against D-galactosamine (GalN)/Lipopolysaccharide (LPS)-induced hepatocyte apoptosis by inhibiting caspase-3 activation, reducing the number of apoptotic cells, and preventing DNA fragmentation. Gomisin A also inhibits caspase-3 activation in CCl4-induced liver injury model mice and enhances MAPK phosphorylation, exerting significant protective effects against liver and kidney damage (Hwang et al., 2013). These findings suggest that Schisandra chinensis mitigates liver injury by modulating apoptosis-related pathways, further highlighting its therapeutic potential.

#### 4.2.4 Autophagy regulation

Autophagy plays a crucial role in liver protection by removing damaged organelles and proteins, thus preventing hepatocyte death (Yang et al., 2024). Schisandrin B has been shown to induce autophagy in Human hepatoma cell line (HepG2) cells, potentially via modulation of the Epidermal growth factor receptor (EGFR)/Phosphatidylinositol 3-Kinase (PI3K)/AKT/Mammalian target of rapamycin (mTOR) signaling pathway in acetaminophen-induced liver injury models (Li et al., 2023c). Zhao et al. demonstrated that Schisandra chinensis essential oil upregulates Microtubule-associated protein 1A/1B-light chain 3-II (LC3-II) and downregulates p62 expression in acetaminophen-overdose models, thereby activating autophagy and promoting liver repair (Zhao et al., 2022). Mass spectrometry-based studies have revealed that Schisandrin A enhances exosome-mediated autophagy, thereby alleviating inflammation and improving symptoms of liver injury in drug-induced liver injury (DILI) models (Li et al., 2023b).

### 4.3 Limitations

Despite strict adherence to the PRISMA guidelines, several limitations still existed. (1) Although the number of studies included was sufficient, the limited data available for subgroup analyses hindered a thorough assessment of Schisandra chinensis' efficacy in liver injury. (2) Selection bias may have influenced the study inclusion process, as studies with positive results are more likely to be published and selected, potentially overestimating the true efficacy of Schisandra chinensis. (3) Significant heterogeneity was observed across studies, likely due to variations in experimental designs, such as animal models, dosage regimens, and treatment durations. This variability compromises the generalizability of the findings and highlights the need for standardized methodologies in future research. (4) Most of the included animal studies employed mouse models. While mouse models represent the most prevalent and well-established approach for liver injury research, they exhibit immunological disparities compared to humans. Therefore, clinical trials are necessary to validate the therapeutic potential and safety profile of Schisandra chinensis and its bioactive compounds.

Despite these limitations, our findings offer valuable insights into the hepatoprotective mechanisms of Schisandra chinensis, providing a strong foundation for future research and clinical applications.

### 4.4 Safety and future prospects

Clinical studies have consistently demonstrated the safety profile of Schisandra chinensis, highlighting its promise as a well-tolerated therapeutic agent. For example, a randomized, double-blind, placebo-controlled trial involving 54 elderly participants reported significant increases in muscle performance without any noted side effects (Cho et al., 2021). Similarly, a study examining its effects on hypertension found no recorded side effects, further confirming its short-term safety and tolerability (Kim et al., 2022).

Beyond its well-established hepatoprotective effects, Schisandra chinensis has demonstrated promising therapeutic potential in other areas, including neuroprotection, cardioprotection, and gut microbiota regulation. These diverse biological activities highlight its versatility as a medicinal herb and suggest its applicability in addressing a wide range of health conditions. However, further research is needed to fully elucidate the pharmacological mechanisms underlying the effects of Schisandra chinensis.

Additionally, a systematic evaluation of its long-term safety and efficacy through rigorous clinical trials is essential to confirm its therapeutic potential and ensure its safe use in diverse populations.

## 5 Conclusion

This systematic review and meta-analysis suggest that Schisandra chinensis exhibits hepatoprotective effects through mechanisms including antioxidant, anti-inflammatory, anti-apoptotic, and autophagy-modulating activities. These findings suggest that Schisandra chinensis could serve as a potential therapeutic agent for liver injury. However, the significant heterogeneity among the included studies may undermine the reliability of these conclusions. To enable clinical application, further high-quality preclinical studies and well-designed clinical trials are necessary to validate its efficacy and clarify the underlying mechanisms of action.

## Data Availability

The raw data supporting the conclusions of this article will be made available by the authors, without undue reservation.

## Glossary

Akt: Protein kinase B
ALP: alkaline phosphatase
ALT: alanine aminotransferase
AMPK: Adenosine 5′-monophosphate-activated protein kinase
Apaf-1: Apoptotic protease activating factor 1
ARE: Antioxidant response element
AST: aspartate aminotransferase
Bax: BCL2-associated X protein
Bcl-2: B-cell lymphoma 2
CCND1: Cyclin D1
CI: confidence interval
COX-2: Cyclooxygenase-2
CYP2E1: Cytochrome P450 proteins
EGFR: Epidermal growth factor receptor
ERK: Extracellular signal-regulated protein kinase
FoxO1: Forkhead box protein O1
GalN: D-galactosamine
GCLM: Glutamate-cysteine Ligase
GSH: glutathione
HepG2: Human hepatoma cell line
HO-1: Heme oxygenase-1
IL-1β: Interleukin-1 beta
IL-6: Interleukin-6
IL-17: Interleukin-17
IL-18: Interleukin-18
iNOS: Inducible nitric oxide synthase
Keap1: Kelch-like ECH-associated protein 1
LC3-II: Microtubule-associated protein 1A/1B-light chain 3-II
LPS: Lipopolysaccharide
MDA: malondialdehyde
mTOR: Mammalian target of rapamycin
MyD88: Myeloid differentiation primary response 88
NF-κB: nuclear factor-κB
NQO1: NAD(P)H quinone dehydrogenase 1
Nrf2: nuclear factor erythroid-2-related factor 2
PCNA: Proliferating cell nuclear antigen
PI3K: Phosphatidylinositol 3-Kinase
PRISMA: Preferred Reporting Items for Systematic Reviews and Meta-Analysis
p-GSK3β: Phospho-Glycogen synthase kinase 3 beta
PXR: pregnane X receptor
ROS: Reactive oxygen species
SCF: Schisandra chinensis Fructus
SD: standard deviation
SEM: standard error of the mean
SMD: standardized mean difference
SOD: superoxide dismutase
SYRCLE: Systematic Review Centre for Laboratory animal Experimentation
TG: Triglyceride
TLR4: Toll-like receptor 4
TNF-α: Tumor necrosis factor-alpha
