# Supplementary material for: Efficacy of Schisandra chinensis in liver injury: a systematic review and preclinical meta-analysis
Source: Front Pharmacol. 2025 Aug 4;16:1627081. doi: 10.3389/fphar.2025.1627081 (PMC12358260; doi:10.3389/fphar.2025.1627081)
Supplement: Supplementary file 1 [file DataSheet1.docx]

**Supplementary Table 1 Literature search strategy**

1. **PubMed Search**

| **NO** | **Search Details** |
| --- | --- |
| #1 | Chemical and Drug Induced Liver Injury"[Mesh] |
| #2 | (((((((((((((((((((((((((((((((((((("Chemical and Drug Induced Liver Injury"[Mesh]) OR (Hepatitis, Toxic)) OR (Toxic Hepatitis)) OR (Hepatitides, Toxic)) OR (Toxic Hepatitides)) OR (Hepatitis, Drug-Induced)) OR (Drug-Induced Hepatitides)) OR (Drug-Induced Hepatitis)) OR (Hepatitides, Drug-Induced)) OR (Hepatitis, Drug Induced)) OR (Drug-Induced Acute Liver Injury)) OR (Drug Induced Acute Liver Injury)) OR (Acute Liver Injury, Drug-Induced)) OR (Acute Liver Injury, Drug Induced)) OR (Liver Injury, Drug-Induced, Acute)) OR (Drug-Induced Liver Disease)) OR (Disease, Drug-Induced Liver)) OR (Diseases, Drug-Induced Liver)) OR (Drug Induced Liver Disease)) OR (Drug-Induced Liver Diseases)) OR (Liver Disease, Drug-Induced)) OR (Liver Diseases, Drug-Induced)) OR (Drug-Induced Liver Injury)) OR (Drug-Induced Liver Injuries)) OR (Drug Induced Liver Injury)) OR (Injuries, Drug-Induced Liver)) OR (Injury, Drug-Induced Liver)) OR (Liver Injuries, Drug-Induced)) OR (Liver Injury, Drug-Induced)) OR (Liver Injury, Drug Induced)) OR (Chemically-Induced Liver Toxicity)) OR (Chemically-Induced Liver Toxicities)) OR (Chemically Induced Liver Toxicity)) OR (Liver Toxicities, Chemically-Induced)) OR (Liver Toxicity, Chemically-Induced)) OR (Toxicities, Chemically-Induced Liver)) OR (Toxicity, Chemically-Induced Liver) |
| #3 | #1 OR #2 |
| #4 | Schisandra[Mesh] |
| #5 | (((((((((("Schisandra"[Mesh]) OR (Gomisi)) OR (Gomisi)) OR (Schisandra chinensis)) OR (Wuweizi)) OR (Wu wei zi)) OR (Schisandra glabra)) OR (Bay Starvine)) OR (Bay Starvines)) OR (Starvine, Bay) OR (schizandro) OR (schizandrin), (schisantherin) OR (gomisin)) |
| #6 | #4 OR #5 |
| #7 | #3 AND #6 (**Results: 246**) |

1. **Embase Search**

| **NO** | **Search Details** |
| --- | --- |
| #1 | ('acute liver injury':ab,ti OR 'blunt liver trauma':ab,ti OR 'hepatic damage':ab,ti OR 'hepatic injury':ab,ti OR 'hepatic lesion':ab,ti OR 'hepatic trauma':ab,ti OR 'injury, liver':ab,ti OR 'liver damage':ab,ti OR 'liver lesion':ab,ti OR 'liver parenchymal injury':ab,ti OR 'liver trauma':ab,ti OR 'liver wound':ab,ti OR 'trauma, hepatic':ab,ti OR 'trauma, liver':ab,ti OR 'wound, liver':ab,ti) AND 'liver injury':ab,ti |
| #2 | chinese schizandra':ab,ti OR 'wu wei zi':ab,ti OR 'schisandra chinensis':ab,ti |
| #3 | #1 AND #2 (**Results: 19**) |

1. **WEB OF SCIENCE**

| **NO** | **Search Details** |
| --- | --- |
| #1 | TS=(Toxic Hepatitis OR Toxic Hepatitides OR Drug-Induced Hepatitides OR Drug-Induced Hepatitis OR Drug-Induced Acute Liver Injury OR Drug Induced Acute Liver Injury OR Drug-Induced Liver Disease OR Chemically-Induced Liver Toxicity OR Chemically-Induced Liver Toxicities OR Chemically Induced Liver Toxicity) |
| #2 | TS=( Schizandra OR Gomisi OR Schisandra chinensis OR Wuweizi OR Wu wei zi OR Schisandra glabra OR Bay Starvine OR Bay Starvines |
| #3 | #1 AND #2(**Results: 116**) |

1. **Cochrane Search**

| **NO** | **Search Details** |
| --- | --- |
| #1 | MeSH descriptor: [Chemical and Drug Induced Liver Injury] explode all trees |
| #2 | Hepatitis, Toxic OR Toxic Hepatitis OR Hepatitides, Toxic OR Toxic Hepatitides OR Hepatitis, Drug-Induced OR Drug-Induced Hepatitides OR Drug-Induced Hepatitis OR Hepatitides, Drug-Induced OR Hepatitis, Drug Induced OR Drug-Induced Acute Liver Injury OR Drug Induced Acute Liver Injury OR Acute Liver Injury, Drug-Induced OR Acute Liver Injury, Drug Induced OR Liver Injury, Drug-Induced, Acute OR Drug-Induced Liver Disease OR Disease, Drug-Induced Liver OR Diseases, Drug-Induced Liver OR Drug Induced Liver Disease OR Drug-Induced Liver Diseases OR Liver Disease, Drug-Induced OR Liver Diseases, Drug-Induced OR Drug-Induced Liver Injury OR Drug-Induced Liver Injuries OR Drug Induced Liver Injury OR Injuries, Drug-Induced Liver OR Injury, Drug-Induced Liver OR Liver Injuries, Drug-Induced OR Liver Injury, Drug-Induced OR Liver Injury, Drug Induced OR Chemically-Induced Liver Toxicity OR Chemically-Induced Liver Toxicities OR Chemically Induced Liver Toxicity OR Liver Toxicities, Chemically-Induced OR Liver Toxicity, Chemically-Induced OR Toxicities, Chemically-Induced Liver OR Toxicity, Chemically-Induced Liver):ti,ab,kw |
| #3 | #1 OR #2 |
| #4 | MeSH descriptor: [Schisandra] explode all trees |
| #5 | (Schisandra OR Gomisi OR Gomisi OR Schisandra chinensis OR Wuweizi OR Wu wei zi OR Schisandra glabra OR Bay Starvine OR Bay Starvines OR Starvine, Bay OR Starvines, Bay):ti,ab,kw |
| #6 | #4 OR #5 |
| #7 | #3 AND #6(**Results: 2**) |

**5 CNKI Search**

| **NO** | **Search Details** | **Results** |
| --- | --- | --- |
| #1 | (SU='五味子' OR SU='北五味子' OR SU='木脂素' OR SU='五味子醇' OR SU='五味子素' OR SU='五味子甲素' OR SU='五味子乙素' OR SU='五味子多糖') AND (SU='肝损伤' OR SU='药物性肝损伤' OR SU='化学性肝损伤' OR SU='酒精性肝损伤' OR SU='肝保护' OR SU='肝毒性') | 234 |

**6 Wanfang** **Search**

| **NO** | **Search Details** | **Results** |
| --- | --- | --- |
| #1 | (主题:("五味子" OR "北五味子" OR "华中五味子" OR "木脂素"OR "五味子醇" OR "五味子甲素" OR "五味子乙素" OR "五味子多糖" OR "五味子醇甲")) AND (主题:("肝损伤" OR "药物性肝损伤" OR "化学性肝损伤" OR "酒精性肝损伤" )) | 205 |

**7 VIP Database Search**

| **NO** | **Search Details** | **Results** |
| --- | --- | --- |
| #1 | (M=("五味子" + "北五味子" + "华中五味子" + "木脂素" + "五味子醇" + "五味子素" + "五味子甲素" + "五味子乙素" + "五味子多糖"))*(M=("肝损伤" + "药物性肝损伤" + "化学性肝损伤" + "酒精性肝损伤" + "肝保护" + "肝毒性")) | 125 |

**8 CBM Search**

| **NO** | **Search Details** |
| --- | --- |
| #1 | "五味子"[常用字段:智能] OR "北五味子"[常用字段:智能] AND "华中五味子"[常用字段:智能] AND "木脂素"[常用字段:智能] AND "五味子醇"[常用字段:智能] AND "五味子素"[常用字段:智能] AND "五味子乙素"[常用字段:智能] AND "五味子多糖"[常用字段:智能] |
| #2 | "肝损伤"[常用字段:智能] OR "药物性肝损伤"[常用字段:智能] OR "化学性肝损伤"[常用字段:智能] OR "酒精性肝损伤"[常用字段:智能] OR "肝保护"[常用字段:智能] OR "肝毒性"[常用字段:智能] |
| #3 | #1 AND #2 (**Results: 163**) |

**Supplementary Table 2. Profile of the examined literature**

1. AST; ②ALT; ③ALP; ④SOD; ⑤ MDA ⑥GSH; ⑦MSH; ⑧ TNF-α; ⑨IL-6; ⑩IL-1β; ⑩CATALASE; ⑪ TG; ⑫ MDA.

| **Author** | **year** | **Species (sex. n = experimental/model group)** | **Model (method)** | **Drug** | **Dosing regimen** | **Mechanisms** | **Outcome** |
| --- | --- | --- | --- | --- | --- | --- | --- |
| Lin | 2024 | Kun-Ming mice; 16/16; 18-22 g; male | Diosbulbin B(300 mg/kg) once; 1 hour after Schisandrin B injection;  intraperitoneal injection | Schisandrin B | 30 mg/kg for once | hibiting CYP3A4-mediated bioactivation | ①②⑤ |
| Chi | 2024 | C57BL/6 mice; 8/8; ?; male |  |  |  | modulating the gut microbiota-tryptophan metabolism-AHR pathway axis |  |
|  |  |  |  |  |  |  |  |
| Ip, S P  et al. | 1996 | Sprague-Dawley;  5/5;240-260g; male | Aflatoxin B (2 mg/kg) / CdCl (20 μmol/kg); once, 24 hours before sacrifice; intraperitoneal injection | Schisandra Chinensis Extract | l.6 g/kg/d for three days | Antioxidant and detoxification | ②⑤⑥ |
| Gao et al. | 2016 | ICR mice;12/12;  18-22g; male | D-Galactosamine (800 mg/kg); once, 12 hours before the last administration; intraperitoneal injection | Schisandrin B | 200 mg/kg three time/d, with an interval of 8 h;? | Heat shock proteins 27 and 70 were liver injury | ② |
| Jiang  et al. | 2015 | C57BL/6 mice;  6/6;20-22g;male | Acetaminophen(400 mg/kg); once, 6 hours before sacrifice;  intraperitoneal injection | Schisandrin A; Schisandrin B; Schisandrin C; Schisandrol A; Schisandrol B | 400 mg/kg/d,  for 7days | Activities of CYP450 isoforms | ①②⑥ |
| Wang  et al. | 2014 | C57BL/6; 8/8; 20-25g; male | Acetaminophen(300 mg/kg); once, 24 hours before sacrifice; intraperitoneal injection | Schisandra Chinensis Extract | 50 mg/kg 3h after acetaminophen treatment | Protection of mitochondria and lysosomes, and inhibition of the phospho-c-Jun N-terminal kinase signaling pathway. | ①②⑥ |
| Li  et al. | 2020 | ICR mice; 10/10; 15-20g; male | Acetaminophen (300 mg/kg); once, after the last administration; intraperitoneal injection | Schisandra Chinensis Extract; Schisandrin B; Schisandrol A; Schisandrol B | ? ; for 3days | Regulation of inflammatory factors, oxidative stress | ①②④⑤⑧ |
| Yuan  et al. | 2018 | ICR mice；10/10; 19-21g; male | 50% Ethanol (12 mL/kg); once, 1 hour after the last administration; intragastric administration | Schisandra chinensis acid polysaccharide | 20 mg/kg; once daily for 15 days | Reduction of  CYP2E1-dependent oxidative stress | ①②④⑤⑪ |
| Shan  et al. | 2019 | ICR mice ;  15/15; ?;male | Concanavalin A (40 mg/kg); once, after the last administration; intravenous injection | Schisandra chinensis polysaccharide | 0.8 mg/10g/d, for 21days | Regulation of Nrf2/ARE and TLR4/NF-κB Signaling Pathway | ①②④⑤⑥⑧⑨⑫ |
| Su et al. | 2019 | Sprague-Dawley;  8/8;  180-220g;male | Alcohol(10 mL/kg); once daily for 2 weeks; intragastric administration | Lignans Extract | 800mg/kg/d, for 2 weeks | Inhibition of CYP2E1 activation and activation of the Nrf2/ARE signaling pathway | ①②⑤⑧⑨⑫ |
| Che et al. | 2019 | ICR mice; 10/10; 20-23g; male | Acetaminophen(250 mg/kg); once, 1 hour after the last administration; intraperitoneal injection | Schisandra chinensis acid polysaccharide | 40 mg/kg/d, for 2 weeks | Inhibition of oxidative stress, inflammation, and cell apoptosis | ①②④⑤⑥⑧⑫ |
| Zhao  et al. | 2022 | C57BL/6 mice;  8/8; 20-22g;male | Acetaminophen (300 mg/kg); once, 24 hours before sacrifice; intraperitoneal injection | Schisandra chinensis essential oil | 2g/kg/d for days | Alleviation of oxidative stress and activation of autophagy | ①②④⑤⑥⑧⑨ |
| Xie et al. | 2014 | Sprague-Dawley; 6/6; 180-220g; male | CCl4 (2 mL/kg); once, 1.5 hours after the after the last administration; intraperitoneal injection | Lignans Extract | 500 mg/kg, 7 times with an interval of 12h | Anti-oxidation way. | ④⑤⑥ |
| Zhai  et al. | 2018 | C57BL/6 mice; 8/8; 20-22g; male | Acetaminophen (300 mg/kg); once, 24 hours before sacrifice; intraperitoneal injection | Schisandra chinensis essential oil | 0.75g/kg, once a day) for 7 consecutive days | Reduction of CAA production and mitigation of CTX-induced toxicity | ①②④⑥ |
| Chen  et al. | 2017 | Sprague-Dawley rats; 9/9; 200-220g; male | Cyclophosphamide (300 mg/kg); once daily for 7 days, 30 minutes after intragastric administration; intravenous injection | Schisandra Chinensis Extract | 0.75g/kg/d for 7 days | Suppression of oxidative stress and modulation of the NF-κB and JNK signaling pathways. | ①②④⑥ |
| Wei et al. | 2019 | Sprague-Dawley rats; 6/6; 210-250g; male | Cyclosporin A (50 mg/kg/day); once daily for 14 days; intragastric administration | Schisandra Chinensis Extract | 200mg/kg; 3 times for 1 day | Activation of the Nrf2 signaling pathway and suppression of apoptosis. | ①② |
| Li et al. | 2014 | ICR mice; 10/10; 18-22g; male | Acetaminophen(400 mg/kg); once, 6 hours before sacrifice; intraperitoneal injection | Schisandrin B | 200mg/kg/d for 7 days | Heat shock proteins 27 and 70 were liver injury | ①②③④⑥⑧⑩ |
| Dai et al. | 2022 | C57BL/6 mice; 6/6; 20-22g; male | Acetaminophen (400 mg/kg); once, 12 hours after the last dose of SinC; intraperitoneal injection | Schisandrin C | 200mg/kg; 7 times at intervals of 12h | Regulation of Nrf2 signaling pathway | ①②④⑥ |
| Jiang  et al. | 2016 | C57BL/6 mice; 6/6; 20-22g; male | Acetaminophen (400 mg/kg); once, 15 minutes after the last gavage of SolB; intraperitoneal injection | Schisandrol B | 200mg/kg; 7 times at intervals of 12h | Activation of the NrF2/ARE pathway and regulation of NRF2 target genes | ①②④⑥ |
| Jiang  et al. | 2015 | C57BL/6 mice; 6/6; 20-22g; male | Acetaminophen (400 mg/kg); once, 15 minutes after the last gavage of SolB; intraperitoneal injection | Schisandrol B | 200mg/kg; 7 times at intervals of 12h | Inhibition of CYP-mediated bioactivation and regulation of liver regeneration | ①②④⑥ |
| Li et al. | 2018 | C57BL/6 mice; 18/18;?; male | alcohol-containing Lieber-DeCarli formulation-based liquid diet (alcohol provided 27.5% of total calories) for four weeks | Schisantherin A | 116mol/kg, daily for 7 days | Regulation of alcohol metabolism and the NF-kB pathway. | ①② |
| Lu  et al. | 2014 | ICR mice; 10/10; 18-22g; male | D-Galactosamine (800 mg/kg/day); 3 times, after the last dose of SchA; intraperitoneal injection | Schisandrin A | 50mg/kg/d for 2 weeks | Activation of autophagy flux and inhibition of apoptosis. | ①②⑥ |
| Yan  et al. | 2021 | C57BL/6 mice; 12/12; 18-22g; male | D-Galactosamine (200 mg/kg); once daily for 8 weeks; subcutaneous injection | Schisandrol A | 30 mg/kg/d for 8 weeks | Anti-oxidative stress and anti-inflammation | ⑤ |
| S-H  et al. | 2008 | ICR mice; 15/15; 20-22 g; male | GalN (700 mg/kg) + LPS (10 µg/kg); intraperitoneal injection; once | Gomisin A | 200 mg/kg;1 hour before D-GalN/LPS injection | Antioxidant activity, anti-apoptotic activity | ①④⑥⑩ |
| Liang  et al. | 2022 | C57BL/6J mice;  6/6 20-24g;male; | lithocholic acid (125 mg/kg); intraperitoneal injection; twice daily; starting from day 4 | Schisandrol B | 100 mg/kg;  twice daily; for 7 days | Inhibition of pyroptosis via PXR activation | ①②③ |
| Wang  et al. | 2014 | ICR mice; 10/10; 20–22g; male | Alcohol (50%); once 1 h later; gastrically infused | Lignans Extract | high-level ; once per day for 60 days | Enhancement of antioxidant status and positive effect of their combination | ①②⑤⑥ |
| Lam  et al. | 2023 | BALB/c mice; 4/4;?; male | Ethanol (35% for 7 days, 40% for the following 7 days, and 52% binge on the 15th day); oral gavage | Schisandrin B | 20 mg/kg; 1 h after ethanol administration for14 days | Regulation of EGFR-mediated activation of autophagy | ①②④⑤⑥ |
| Chen  et al. | 2023 | C57BL/6J mice; 4/4; 18-22g; male | 10% CCl4; 3 times per week for 4 weeks; intraperitoneal injection | Schisandrin C | 50 mg/kg; once daily for 4 weeks | Regulation of NF-κB and p38/ERK MAPK signaling pathways | ①② |
| Shi et al. | 2022 | Sprague-Dawley rat; 10/10; 180-200g; male | Pirarubicin, 3 mg/kg/day; once a week for 8 weeks; intravenous injection | Schisandrin B | 50 mg/kg/day; administered for 8 weeks | Inhibition of oxidative stress and ferroptosis | ①②④⑤⑥ |
| Nagappan et al. | 2018 | C57BL/6J mice; 10/10; 18-22g; male | Ethanol(5%) chronic-binge feeding for 10 days; single dose of 5 g/kg orally on the 11th day | Gomisin N | 20 mg/kg; daily for 10 days | Inhibition of oxidative stress for reduction of ferroptosis and lipid peroxidation | ①②④⑤⑥⑩⑪ |
| Chang  et al. | 2023 | Kunming mice; 6/6; 17-20 g; male | Dictamnine, 200 mg/kg; once 24 hours before sacrifice; intraperitoneal injection | Schisantherin A | 20 mg/kg; pretreatment 1 hour before DIC administration | Inhibition of CYP3A-mediated metabolic activation | ①②⑤ |
| Teraoka et al. | 2012 | Wistar rat; 8/8; 150-200g; male | 5 mL/kg (50% CCl₄ in olive oil); single intragastric injection; 36 hours before sacrifice | Gomisin A | 100 mg/kg; intraperitoneal injection at 30 min and 24 h before CCl₄ | Inhibition of oxidative stress and NF-κB activation, | ①②⑤ |
| Xu et al. | 2014 | Wistar rats; 25/25; 180-200g; male | 50% ethanol; 7 mL/kg; intragastric; 10 days | Schisandra lignans,  Schisandra polysaccharides | 100 mg/kg; 4 days | Antioxidant and Anti-inflammation | ①②④⑤ |
| Li et al. | 2014 | ICR mice; 10/10; 18-22g; male | 50% ethanol; 12 mL/kg; intragastric administration; single dose; sacrificed 1 hour later | Schisandra polysaccharides | 100 mg/kg; daily; for 15 days | Antioxidant | 1. ②⑪ |
| Chen  et al. | 2014 | ICR mice; 10/10; 18-22g; male | 10% CCl₄ 2.0 mL/kg; single intraperitoneal injection; 6 hours before sacrifice | Schisandra polysaccharides | 100 mg/kg; daily; for 15 days | Antioxidant | ①②⑤⑥ |
| Wu et al. | 2014 | Wistar rats; 8/8;  180–200g; male | high-fat chow feeding for 16 weeks | Schisandra polysaccharides | 50 mg/kg; daily; for 12 weeks | Hepatoprotection | 1. ② |
| Wang  et al. | 2014 | ICR mice; 10/10; 20–22g; male | 50% ethanol; single intragastric injection; 12 hours before sacrifice | Schisandra polysaccharides | 50 mg/kg; daily;for 15 days | Improvement of antioxidative capacity | ①② |
| Xu et al. | 2011 | Wistar rats; n = 15/15; 180-220g; male | 5 mL/kg (10% CCl₄ in plant oil); single intragastric injection; 3 days before sacrifice | Schisandra polysaccharides | 400 mg/kg; daily; for 1 week | Antagonism of CCl₄-induced lipid peroxidation | 1. ②⑤ |
| Zhou  et al. | 2018 | Kunming mice; 6/6,18–22 g; male | 5 mL/kg (50% CCl₄ in olive oil); single intragastric injection; 36 hours before sacrifice | Schisandrae Chinensis Fructus extract | 215 mg/kg; daily; for 8weeks | improvement of antioxidative capacity | ①②⑤ |
| Yao et al. | 2014 | Kunming mice; 10/10,18–22 g; male | 0.2% CCl₄; 10 mL/kg; single intraperitoneal injection; mice fasted for 22 h before sacrifice. | Schisandra lignans | 200 mg/kg; dailye; for 8 days | Improvement of antioxidant capacity | ①② |
| Li et al. | 2013 | ICR mice; 12/12; 18-22 g; male; | 25 mg/kg  Concanavalin A in saline; single intravenous injection; 6 hours before sacrifice | Schisandrol B | 200 mg/kg; 3 times in 24 hours | Induction of heat shock proteins HSP27 and HSP70 | ①② |
| Dai et al. | 2022 | C57BL/6 mice; 6/6; 20–22 g; male; 6/6; 20–22 g | 400 mg/kg Acetaminophen; single intraperitoneal injection; 12 hours before sacrifice | Schisandrin C | 200 mg/kg; daily; for 7 days | Regulation of the Nrf2 signaling pathway, reduction of CYP2E1 expression | ①②③④⑤⑥⑧ |
| Wang  et al. | 2020 | ICR mice; 12/12; 18-22 g;male | 2.25 mg Bacillus Calmette-Guerin and 80 ug lipopolysaccharide; intraperitoneal injection; 8 hours before sacrifice | Schisandra lignans | 0.2 mg/10 g; daily; for 12 days | Improvement of antioxidative capacity | ①②⑤⑥ |
| Xu et al. | 2011 | Wistar rats; n = 15/15; 200±20 g; male | 5 mL/kg (10% CCl₄ in plant oil); single intragastric injection; 3 days before sacrifice | Schisandra polysaccharides | 400 mg/kg; daily; for 1 week | Antagonism of CCl₄-induced lipid peroxidation | 1. ②⑤ |
| Guo  et al. | 2024 | ICR mice; 10/10; 20-22 g; male | 3% DSS solution;  oral administration for 7 days; 3 weeks before sacrifice | Schisandra polysaccharides | 200 mg/kg; daily; for 3 weeks | Improvement of antioxidant capacity | ①②④⑥⑧ |
| Che et al. | 2019 | ICR mice; 10/10; 19-22 g; male | 250 mg/kg Acetaminophen; single intraperitoneal injection; 24 hours before sacrifice | Schisandra polysaccharides | 100 mg/kg; daily; for 2 weeks. | Improvement of antioxidative capacity, inhibition of hepatocyte apoptosis | ①②⑤⑥ |
| Sun et al. | 2019 | Kunming mice; 10/10;  20-22g;male | 10 mL/kg (0.12% CCl₄ in olive oil); single intraperitoneal injection; 12 hours before sacrifice | Schisandra polysaccharides | 200 mg/kg; daily; for 10 days | Inhibition of inflammation | 1. ②⑧⑨ |
| Wang  et al. | 2020 | ICR mice; 10/10; 18–22 g; male | 3 mL/kg  (50% CCl₄ in olive oil); subcutaneous injection; twice a week for 6 weeks | Schisandrin A | 40 mg/kg; once a day; for 6 weeks | Regulation of NLRP3/NF-κB and TGF-β/Smad3signaling pathways | 1. ② |
| Wang  et al. | 2019 | ICR mice; 10/10; 18–20 g; male | 10 mL/kg (0.3% CCl₄ in peanut oil); intraperitoneal injection; 16 hours before sacrifice | Schisandrin A | 400 mg/kg; twice daily; for 7 days | Improvement of antioxidative capacity | ①②④⑤⑧ |
| An et al. | 2014 | Sprague Dawley rats; 6/6; 180–250 g; male | 2.5 mg/kg doxorubicin; intraperitoneal injection; once a week for 5 weeks | Schisandra lignans | 100 mg/kg; once daily; for 30 days | Improvement of antioxidative capacity and anti-inflammatory  effects | ①②⑧⑩ |
| Sun et al. | 2021 | Wistar rats; 6/6; 180-200 g; male | 20% CCl₄  (2 mL/kg in olive oil);  intraperitoneal injection; three times per week for 8 weeks | Schisandrin B | 50 mg/kg; daily; for 4 weeks | Inhibition of NF-kB/COX-2 activation | ①② |
| Zhu et al. | 2012 | Sprague Dawley rats; 10/10; 180-200 g; male | 100 mg/kg (sodium valproate); intragastric administration; daily for 2 weeks | Schisandrin B | 200 mg/kg; daily; for 2 weeks | Clearance of free radicals and inhibition of lipid peroxidation | ①②③ |
| Qiu et al. | 2018 | Sprague Dawley rats; 21/21; 180-200 g; male | 500 mg/kg Acetaminophen; single intragastric injection; 24 hours before sacrifice | Schisandrae Chinensis Fructus extract | 2.0 g/kg; daily; for 21 days | Activation of Nrf2 signaling pathway | ①②④⑤ |
| Yan et al. | 2009 | Kunming mice; 10/10; 18–20 g; male | 500 mg/kg Acetaminophen; intraperitoneal injection; 24 hours before sacrifice | Schisandrae Chinensis Fructus extract | 270 mg/kg; once daily; for 5 days | Synergistic enhancement of antioxidant capacity and reduction of oxidative stress | ①② |
| Wang  et al. | 2019 | ICR mice; 20/20; 20–22 g; male | 50% ethanol solution (10 mL/kg); single intragastric injection; 16 hours before sacrifice | Schisandra polysaccharides | 100mg/kg; daily; for14 days | Improvement of antioxidative capacity and reduction of oxidative stress | ①② |
| Zhao et al. | 2006 | ICR mice; 14/14; 20–22 g; male | Alcohol liquid diet + LPS (1 mg/kg intravenous injection); 8 weeks feeding followed by LPS injection; 6 hours before sacrifice | Schisandrae Chinensis Fructus extract | 1.5%; daily;  for 8 weeks | Improvement of antioxidative capacity and reduction of oxidative stress | ①② |

Note：①AST; ②ALT; ③ALP; ④SOD; ⑤ MDA ⑥GSH; ⑦MSH; ⑧ TNF-α; ⑨IL-6; ⑩IL-1β; ⑩CATALASE; ⑪ TG; ⑫ MDA.

**Supplementary Table 3. The methodological quality of included studies.**

| Study | A | B | C | D | E | F | G | H | I | J | Score |
| --- | --- | --- | --- | --- | --- | --- | --- | --- | --- | --- | --- |
| Ip 1996 | ？ | ？ | ？ | √ | ？ | ？ | √ | √ | √ | √ | **5** |
| Gao 2016 | ？ | ？ | ？ | √ | ？ | ？ | √ | √ | √ | √ | **5** |
| Jiang 2015 | ？ | ？ | ？ | √ | ？ | ？ | √ | √ | √ | √ | **5** |
| Wang 2014 | ？ | ？ | √ | ？ | ？ | ？ | √ | √ | √ | √ | **5** |
| Li 2020 | ？ | ？ | ？ | √ | ？ | ？ | √ | √ | √ | √ | **5** |
| Yuan 2018 | ？ | ？ | ？ | √ | ？ | ？ | √ | √ | √ | √ | **5** |
| Wang 2014 | ？ | ？ | ？ | √ | ？ | ？ | √ | √ | √ | √ | **5** |
| Shan 2019 | ？ | ？ | ？ | √ | ？ | ？ | √ | √ | √ | √ | **5** |
| Su 2019 | ？ | ？ | ？ | √ | ？ | ？ | √ | √ | √ | √ | **5** |
| Che 2019 | ？ | ？ | ？ | √ | ？ | ？ | √ | √ | √ | √ | **5** |
| Zhao 2022 | ？ | ？ | √ | √ | ？ | ？ | √ | √ | √ | √ | **6** |
| Xie 2014 | ？ | ？ | ？ | √ | ？ | ？ | √ | √ | √ | √ | **5** |
| Zhai 2018 | ？ | ？ | ？ | √ | ？ | ？ | √ | √ | √ | √ | **5** |
| Chen 2017 | ？ | ？ | ？ | √ | ？ | ？ | √ | √ | √ | √ | **5** |
| Wei 2019 | ？ | ？ | √ | √ | ？ | ？ | √ | √ | √ | √ | **6** |
| Li 2014 | ？ | ？ | ？ | ？ | ？ | ？ | √ | √ | √ | √ | **4** |
| Dai 2022 | ？ | ？ | ？ | √ | ？ | ？ | √ | √ | √ | √ | **5** |
| Jiang 2016 | ？ | ？ | ？ | √ | ？ | ？ | √ | √ | √ | √ | **5** |
| Jiang 2015 | ？ | ？ | √ | √ | ？ | ？ | √ | √ | √ | √ | **6** |
| Lu 2014 | ？ | ？ | ？ | √ | ？ | ？ | √ | √ | √ | √ | **5** |
| Yan 2021 | ？ | ？ | ？ | √ | ？ | ？ | √ | √ | √ | √ | **5** |
| S-H 2008 | ？ | ？ | ？ | √ | ？ | ？ | √ | √ | √ | √ | **5** |
| Liang2022 | ？ | ？ | ？ | √ | ？ | ？ | √ | √ | √ | √ | **5** |
| Wang 2014 | ？ | ？ | ？ | √ | ？ | ？ | √ | √ | √ | √ | **5** |
| Lam 2023 | ？ | ？ | ？ | √ | ？ | ？ | √ | √ | √ | √ | **5** |
| Chen 2023 | ？ | ？ | ？ | √ | ？ | ？ | √ | √ | √ | √ | **5** |
| Shi 2022 | ？ | ？ | ？ | √ | ？ | ？ | √ | √ | √ | √ | **5** |
| Nagappan2018 | ？ | ？ | ？ | √ | ？ | ？ | √ | √ | √ | √ | **5** |
| Chang 2023 | ？ | ？ | ？ | √ | ？ | ？ | √ | √ | √ | √ | **5** |
| Teraoka 2012 | ？ | ？ | ？ | √ | ？ | ？ | √ | √ | √ | √ | **5** |
| S-H 2008 | ？ | ？ | ？ | √ | ？ | ？ | √ | √ | √ | √ | **5** |
| Liang2022 | ？ | ？ | ？ | √ | ？ | ？ | √ | √ | √ | √ | **5** |
| Wang 2014 | ？ | ？ | ？ | √ | ？ | ？ | √ | √ | √ | √ | **5** |
| Lam 2023 | ？ | ？ | ？ | √ | ？ | ？ | √ | √ | √ | √ | **5** |
| Xu 2014 | ？ | ？ | ？ | √ | ？ | ？ | √ | √ | √ | √ | **5** |
| Xu 2014 | ？ | ？ | ？ | √ | ？ | ？ | √ | √ | √ | √ | **5** |
| Li 2014 | ？ | ？ | ？ | √ | ？ | ？ | √ | √ | √ | √ | **5** |
| Chen 2014 | ？ | ？ | √ | √ | ？ | ？ | √ | √ | √ | √ | **6** |
| Wu 2014 | ？ | ？ | ？ | √ | ？ | ？ | √ | √ | √ | √ | **5** |
| Wang 2014 | ？ | ？ | ？ | √ | ？ | ？ | √ | √ | √ | √ | **5** |
| Xu 2011 | ？ | ？ | ？ | √ | ？ | ？ | √ | √ | √ | √ | **5** |
| Zhou 2018 | ？ | ？ | ？ | √ | ？ | ？ | √ | √ | √ | √ | **5** |
| Yao 2014 | ？ | ？ | ？ | √ | ？ | ？ | √ | √ | √ | √ | **5** |
| Li 2013 | ？ | ？ | ？ | √ | ？ | ？ | √ | √ | √ | √ | **5** |
| Dai 2022 | ？ | ？ | ？ | √ | ？ | ？ | √ | √ | √ | √ | **5** |
| Wang 2020 | ？ | ？ | ？ | √ | ？ | ？ | √ | √ | √ | √ | **5** |
| Wang 2020 | ？ | ？ | ？ | √ | ？ | ？ | √ | √ | √ | √ | **5** |
| Xu 2011 | ？ | ？ | ？ | √ | ？ | ？ | √ | √ | √ | √ | **5** |
| Guo 2024 | ？ | ？ | ？ | √ | ？ | ？ | √ | √ | √ | √ | **5** |
| Che 2019 | ？ | ？ | ？ | √ | ？ | ？ | √ | √ | √ | √ | **5** |
| Sun 2019 | ？ | ？ | ？ | √ | ？ | ？ | √ | √ | √ | √ | **5** |
| Wang 2020 | ？ | ？ | ？ | √ | ？ | ？ | √ | √ | √ | √ | **5** |
| Wang 2019 | ？ | ？ | ？ | √ | ？ | ？ | √ | √ | √ | √ | **5** |
| An 2014 | ？ | ？ | ？ | √ | ？ | ？ | √ | √ | √ | √ | **5** |
| Sun 2021 | ？ | ？ | ？ | √ | ？ | ？ | √ | √ | √ | √ | **5** |
| Zhu 2012 | ？ | ？ | ？ | √ | ？ | ？ | √ | √ | √ | √ | **5** |
| Qiu 2018 | ？ | ？ | ？ | ？ | ？ | ？ | √ | √ | √ | √ | **4** |
| Yan 2009 | ？ | ？ | ？ | √ | ？ | ？ | √ | √ | √ | √ | **5** |
| Wang 2019 | ？ | ？ | ？ | √ | ？ | ？ | √ | √ | √ | √ | **5** |
| Zhao 2006 | ？ | ？ | ？ | √ | ？ | ？ | √ | √ | √ | √ | **5** |
| NOTE: A, sequence generation; B, baseline characteristics; C, allocation concealment; D, random housing; E, blinding (caregivers/investigators); F, random random for outcome assessment; G, blinding (outcome assessor); H, incomplete outcome data; I, selective outcome reporting; J, other biases | | | | | | | | | | | |

**Supplementary table 4. sesitivity analysis of AST**

| **Study omitted** | **Estimate** | **95% Conf. Interval** |
| --- | --- | --- |
| Jiang 2015 | 2.10 | -2.37,-2.56 |
| Jiang 2015 | 2.10 | -2.36,-2.56 |
| Jiang 2015 | 2.20 | -2.37,-2.56 |
| Jiang 2015 | 2.13 | -2.37,-2.56 |
| Jiang 2015 | 2.10 | -2.37,-2.56 |
| Wang 2014 | 2.09 | -2.36,-2.56 |
| Li 2020 | 2.08 | -2.36,-2.56 |
| Li 2020 | 2.04 | -2.35,-2.55 |
| Li 2020 | 2.17 | -2.42,-2.62 |
| Li 2020 | 2.08 | -2.35,-2.55 |
| Yuan 2018 | 2.12 | -2.39,-2.59 |
| Shan 2019 | 2.08 | -2.34,-2.53 |
| Su 2019 | 2.14 | -2.36,-2.56 |
| Che 2019 | 2.04 | -2.41,-2.61 |
| Zhao 2022 | 2.07 | -2.36,-2.56 |
| Zhai 2018 | 2.07 | -2.36,-2.55 |
| Li 2018 | 2.37 | -2.45,-2.66 |
| Chen 2017 | 2.15 | -2.39,-2.59 |
| Wei 2019 | 2.13 | -2.38,-2.57 |
| Dai 2022 | 2.19 | -2.37,-2.56 |
| Jiang 2016 | 2.10 | -2.36,-2.56 |
| Jiang 2015 | 2.28 | -2.38,-2.58 |
| Lu 2014 | 2.16 | -2.35,-2.55 |
| Liang2022 | 2.12 | -2.38,-2.58 |
| Wang 2014 | 2.10 | -2.39,-2.59 |
| Lam 2023 | 2.10 | -2.43,-2.63 |
| Chen 2023 | 2.20 | -2.39,-2.59 |
| Shi 2022 | 2.13 | -2.37,-2.56 |
| Nagappan 2018 | 2.10 | -2.35,-2.55 |
| Teraoka 2012 | 2.09 | -2.37,-2.56 |
| Xu 2014 | 2.08 | -2.34,-2.53 |
| Xu 2014 | 2.04 | -2.35,-2.54 |
| Chen 2014 | 2.17 | -2.46,-2.66 |
| Wu 2014 | 2.08 | -2.35,-2.55 |
| Wang 2014 | 2.12 | -2.42,-2.62 |
| Chen 2016 | 2.08 | -2.38,-2.58 |
| Xu 2011 | 2.14 | -2.34,-2.53 |
| Zhou 2018 | 2.04 | -2.39,-2.59 |
| Yao 2014 | 2.07 | -2.52,-2.71 |
| Li 2013 | 2.07 | -2.35,-2.55 |
| Dai 2022 | 2.37 | -2.38,-2.58 |
| Wang 2020 | 2.15 | -2.48,-2.68 |
| Wang 2020 | 2.13 | -2.47,-2.67 |
| Xu 2011 | 2.19 | -2.34,-2.53 |
| Guo 2024 | 2.10 | -2.35,-2.55 |
| Che 2019 | 2.28 | -2.37,-2.57 |
| Sun 2019 | 2.16 | -2.39,-2.59 |
| Wang 2020 | 2.12 | -2.38,-2.57 |
| Wang 2019 | 2.10 | -2.36,-2.55 |
| An 2014 | 2.10 | -2.35,-2.55 |
| Sun 2021 | 2.20 | -2.37,-2.56 |
| Zhu 2012 | 2.13 | -2.36,-2.55 |
| Qiu 2018 | 2.10 | -2.35,-2.54 |
| Yan 2009 | 2.09 | -2.43,-2.63 |
| Wang 2019 | 2.08 | -2.32,-2.52 |
| Zhao 2006 | 2.04 | -2.59,-2.8 |

**Supplementary table 5. Sensitivity analysis of ALT**

| **Study omitted** | **Estimate** | **[95% Conf. Interval]** |
| --- | --- | --- |
| Ip 1996 | -2.64 | -2.84, -2.44 |
| Ip1996 | -2.67 | -2.87, -2.47 |
| Gao 2016 | -2.61 | -2.81, -2.41 |
| Jiang 2015 | -2.63 | -2.83, -2.43 |
| Jiang 2015 | -2.63 | -2.83, -2.43 |
| Jiang 2015 | -2.63 | -2.83, -2.43 |
| Jiang 2015 | -2.63 | -2.83, -2.43 |
| Jiang 2015 | -2.63 | -2.83, -2.43 |
| Wang 2014 | -2.62 | -2.82, -2.42 |
| Li 2020 | -2.62 | -2.81, -2.42 |
| Li 2020 | -2.62 | -2.81, -2.42 |
| Li 2020 | -2.62 | -2.81, -2.42 |
| Li 2020 | -2.62 | -2.81, -2.42 |
| Yuan 2018 | -2.67 | -2.87, -2.47 |
| Shan 2019 | -2.62 | -2.82, -2.42 |
| Su 2019 | -2.62 | -2.82, -2.42 |
| Che 2019 | -2.66 | -2.86, -2.46 |
| Zhao 2022 | -2.63 | -2.83, -2.44 |
| Zhai 2018 | -2.62 | -2.82, -2.42 |
| Chen 2017 | -2.74 | -2.94, -2.54 |
| Wei 2019 | -2.63 | -2.83, -2.43 |
| Li 2014 | -2.62 | -2.82, -2.43 |
| Jiang 2016 | -2.63 | -2.83, -2.43 |
| Jiang 2015 | -2.66 | -2.86, -2.46 |
| Li 2018 | -2.74 | -2.94, -2.53 |
| Lu 2014 | -2.62 | -2.81, -2.42 |
| S-H 2008 | -2.61 | -2.81, -2.42 |
| Liang2022 | -2.63 | -2.83, -2.43 |
| Wang 2014 | -2.68 | -2.88, -2.48 |
| Lam 2023 | -2.65 | -2.85, -2.45 |
| Chen 2023 | -2.63 | -2.83, -2.43 |
| Shi 2022 | -2.62 | -2.82, -2.42 |
| Nagappan 2018 | -2.62 | -2.82, -2.42 |
| Teraoka 2012 | -2.62 | -2.82, -2. 43 |
| Xu 2014 | -2.58 | -2.79, -2.38 |
| Xu 2014 | -2.61 | -2.81, -2.41 |
| Li 2014 | -2.72 | -2.92, -2.52 |
| Wu 2014 | -2.62 | -2.81, -2.42 |
| Wang 2014 | -2.72 | -2.92, -2.52 |
| Chen 2014 | -2.65 | -2.85, -2.45 |
| Xu 2011 | -2.61 | -2.81, -2.42 |
| Zhou 2018 | -2.63 | -2.83, -2.43 |
| Yao 2014 | -2.62 | -2.82, -2.42 |
| Li 2013 | -2.61 | -2.81, -2.41 |
| Wang 2020 | -2.77 | -2.97, -2.56 |
| Wang 2020 | -2.72 | -2.93, -2.52 |
| Xu 2011 | -2.61 | -2.81, -2.42 |
| Guo 2024 | -2.65 | -2.85, -2.45 |
| Che 2019 | -2.63 | -2.83, -2.43 |
| Sun 2019 | -2.68 | -2.88, -2.48 |
| Wang 2020 | -2.63 | -2.83, -2.44 |
| Wang 2019 | -2.63 | -2.83, -2.43 |
| Sun 2021 | -2.73 | -2.93, -2.53 |
| Zhu 2012 | -2.62 | -2.81, -2.42 |

**Supplementary table 6.** **Sensitivity analysis of SOD**

| **Study omitted** | **Estimate** | **95% Conf. Interval** |
| --- | --- | --- |
| Ip, S P 1996 | -2.10 | -2.1, -2.32 |
| Ip, S P 1996 | -2.12 | -2.12, -2.34 |
| Li 2020 | -2.09 | -2.09, -2.32 |
| Li 2020 | -2.07 | -2.07, -2.3 |
| Li 2020 | -2.07 | -2.07, -2.3 |
| Li 2020 | -2.07 | -2.07, -2.3 |
| Yuan 2018 | -2.16 | -2.16, -2.39 |
| Shan 2019 | -2.04 | -2.04, -2.26 |
| Su 2019 | -2.07 | -2.07, -2.29 |
| Che 2019 | -2.11 | -2.11, -2.34 |
| Zhao 2022 | -2.08 | -2.08, -2.3 |
| Xie 2014 | -2.16 | -2.16, -2.38 |
| Yan 2021 | -2.08 | -2.08, -2.31 |
| Wang 2014 | -2.09 | -2.09, -2.32 |
| Shi 2022 | -2.10 | -2.1, -2.32 |
| Nagappan 2018 | -2.18 | -2.18, -2.41 |
| Chang 2023 | -2.17 | -2.17, -2.39 |
| Xu 2014 | -2.03 | -2.03, -2.26 |
| Xu 2014 | -2.08 | -2.08, -2.3 |
| Wang 2014 | -2.07 | -2.07, -2.3 |
| Chen 2016 | -2.13 | -2.13, -2.36 |
| Xu 2011 | -2.04 | -2.04, -2.26 |
| Yao 2014 | -2.16 | -2.16, -2.39 |
| Dai 2022 | -2.09 | -2.09, -2.31 |
| Wang 2020 | -2.19 | -2.19, -2.42 |
| Wang 2020 | -2.18 | -2.18, -2.41 |
| Xu 2011 | -2.04 | -2.04, -2.26 |
| Guo 2024 | -2.08 | -2.08, -2.3 |
| Che 2019 | -2.09 | -2.09, -2.32 |
| Wang 2019 | -2.06 | -2.06, -2.29 |
| Qiu 2018 | -2.21 | -2.21, -2.45 |

**Supplementary table 7. Sensitivity analysis of MDA**

| **Study omitted** | **Estimate** | **95% Conf. Interval** |
| --- | --- | --- |
| Ip, S P 1996 | -2.10 | -2.32, -1.87 |
| Ip, S P 1996 | -2.12 | -2.34, -1.89 |
| Li 2020 | -2.09 | -2.32, -1.86 |
| Li 2020 | -2.07 | -2.3, -1.85 |
| Li 2020 | -2.07 | -2.3, -1.85 |
| Li 2020 | -2.07 | -2.3, -1.85 |
| Yuan 2018 | -2.16 | -2.39, -1.93 |
| Shan 2019 | -2.04 | -2.26, -1.82 |
| Su 2019 | -2.07 | -2.29, -1.85 |
| Che 2019 | -2.11 | -2.34, -1.88 |
| Zhao 2022 | -2.08 | -2.3, -1.86 |
| Xie 2014 | -2.16 | -2.38, -1.93 |
| Yan 2021 | -2.08 | -2.31, -1.86 |
| Wang 2014 | -2.09 | -2.32, -1.87 |
| Shi 2022 | -2.10 | -2.32, -1.87 |
| Nagappan 2018 | -2.18 | -2.41, -1.95 |
| Chang 2023 | -2.17 | -2.39, -1.94 |
| Xu 2014 | -2.03 | -2.26, -1.81 |
| Xu 2014 | -2.08 | -2.3, -1.86 |
| Wang 2014 | -2.07 | -2.3, -1.85 |
| Chen 2016 | -2.13 | -2.36, -1.91 |
| Xu 2011 | -2.04 | -2.26, -1.82 |
| Yao 2014 | -2.16 | -2.39, -1.93 |
| Dai 2022 | -2.09 | -2.31, -1.86 |
| Wang 2020 | -2.19 | -2.42, -1.96 |
| Wang 2020 | -2.18 | -2.41, -1.95 |
| Xu 2011 | -2.04 | -2.26, -1.82 |

**Supplementary table 8. Sensitivity analysis of GSH**

| **Study omitted** | **Estimate** | **95% Conf. Interval** |
| --- | --- | --- |
| Ip 1996 | 2.10 | 1.82, 2.37 |
| Ip 1996 | 2.10 | 1.82, 2.37 |
| Jiang 2015 | 2.20 | 1.92, 2.48 |
| Jiang 2015 | 2.13 | 1.85, 2.4 |
| Jiang 2015 | 2.10 | 1.82, 2.37 |
| Jiang 2015 | 2.09 | 1.82, 2.37 |
| Wang 2014 | 2.08 | 1.8, 2.35 |
| Shan 2019 | 2.04 | 1.76, 2.32 |
| Che 2019 | 2.17 | 1.89, 2.46 |
| Zhao 2022 | 2.08 | 1.8, 2.35 |
| Xie 2014 | 2.12 | 1.84, 2.4 |
| Li 2014 | 2.08 | 1.81, 2.35 |
| Jiang 2015 | 2.14 | 1.86, 2.42 |
| S-H 2008 | 2.04 | 1.76, 2.32 |
| Wang 2014 | 2.07 | 1.79, 2.34 |
| Shi 2022 | 2.07 | 1.8, 2.35 |
| Nagappan 2018 | 2.37 | 2.08, 2.65 |
| Li 2014 | 2.15 | 1.87, 2.43 |
| Wang 2014 | 2.13 | 1.85, 2.41 |
| Chen 2016 | 2.19 | 1.91, 2.48 |
| Dai 2022 | 2.10 | 1.83, 2.38 |
| Wang 2020 | 2.28 | 2,2. 2.57 |
| Wang 2020 | 2.16 | 1.87, 2.44 |
| Che 2019 | 2.12 | 1.84, 2.4 |


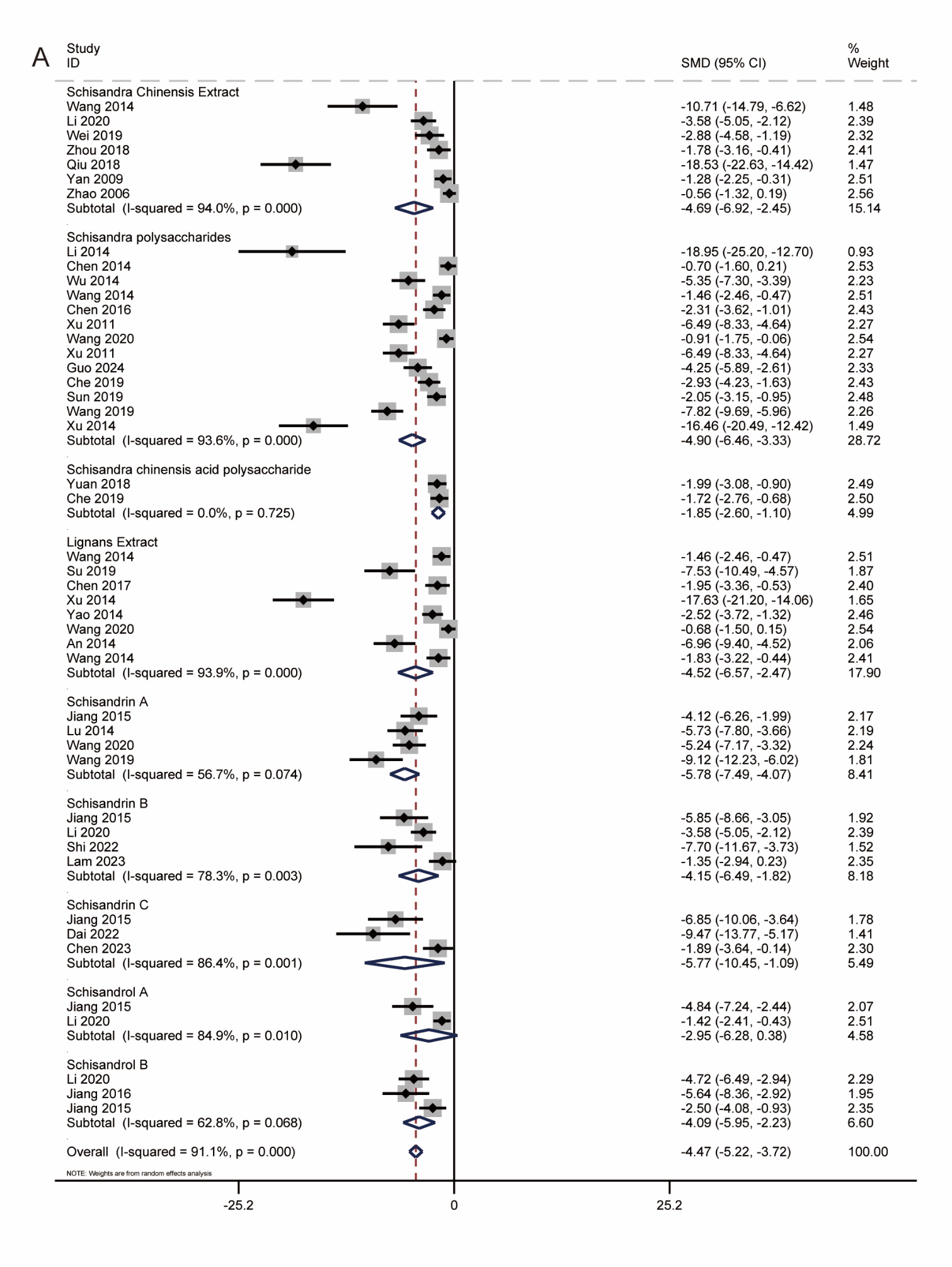


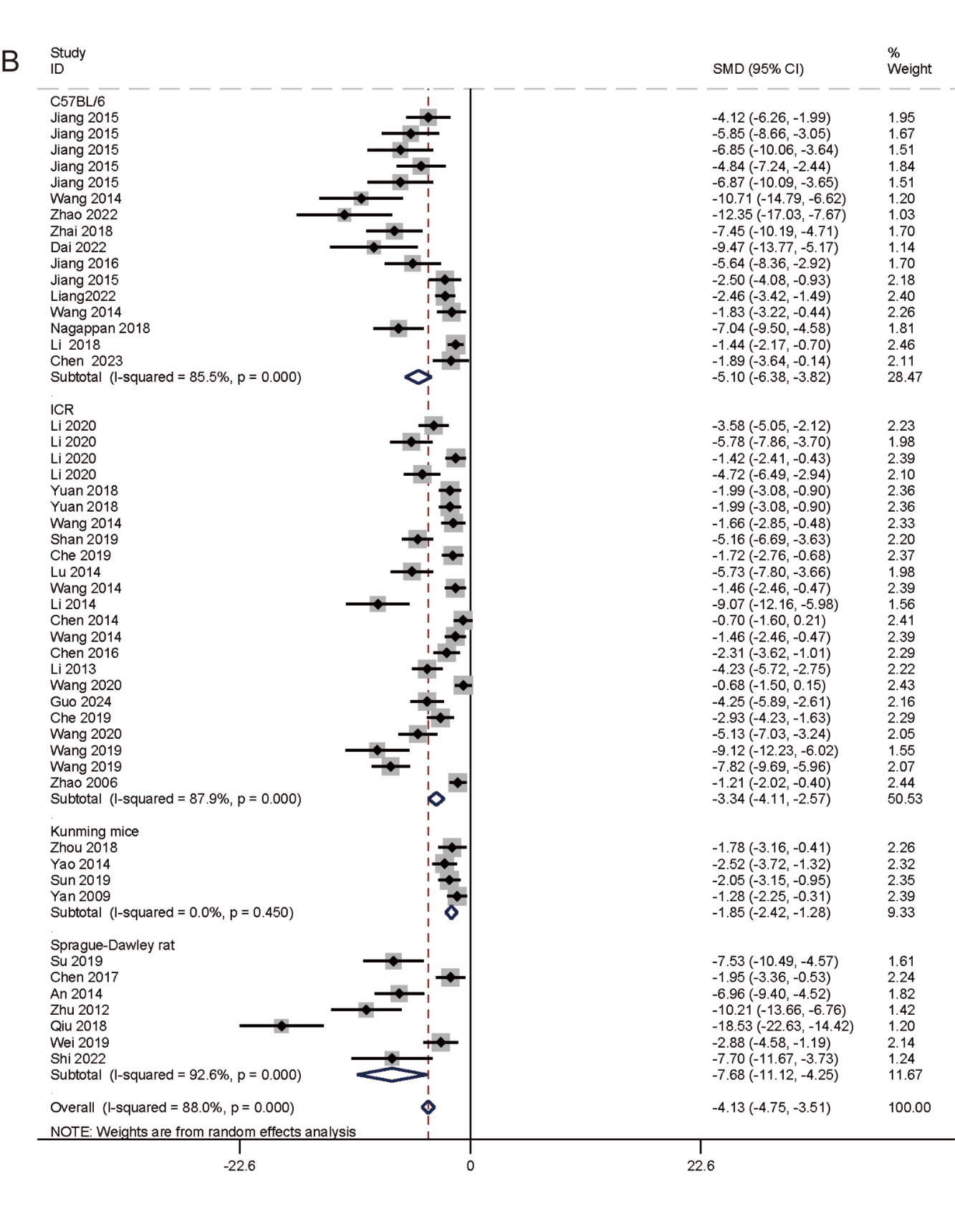


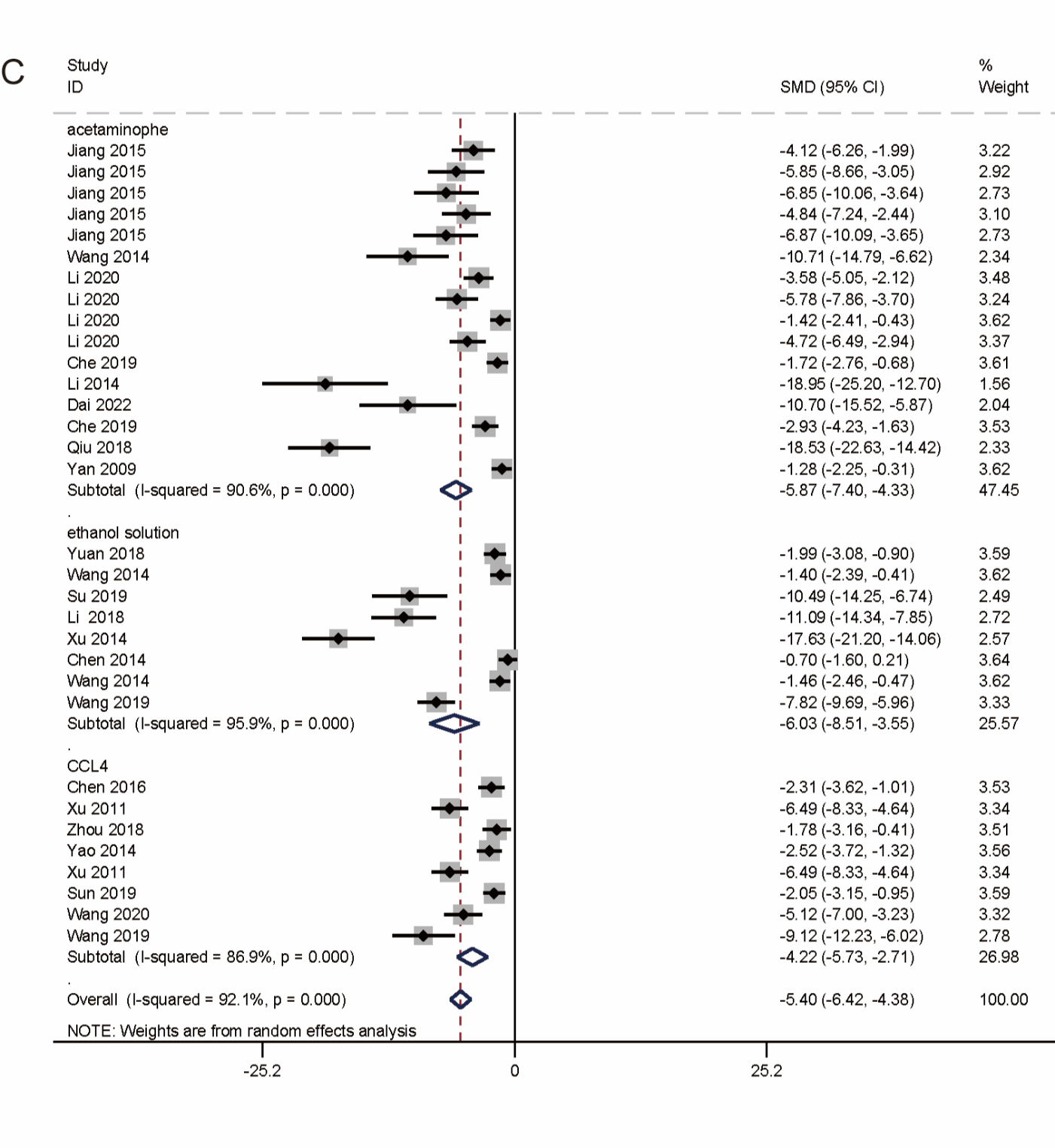


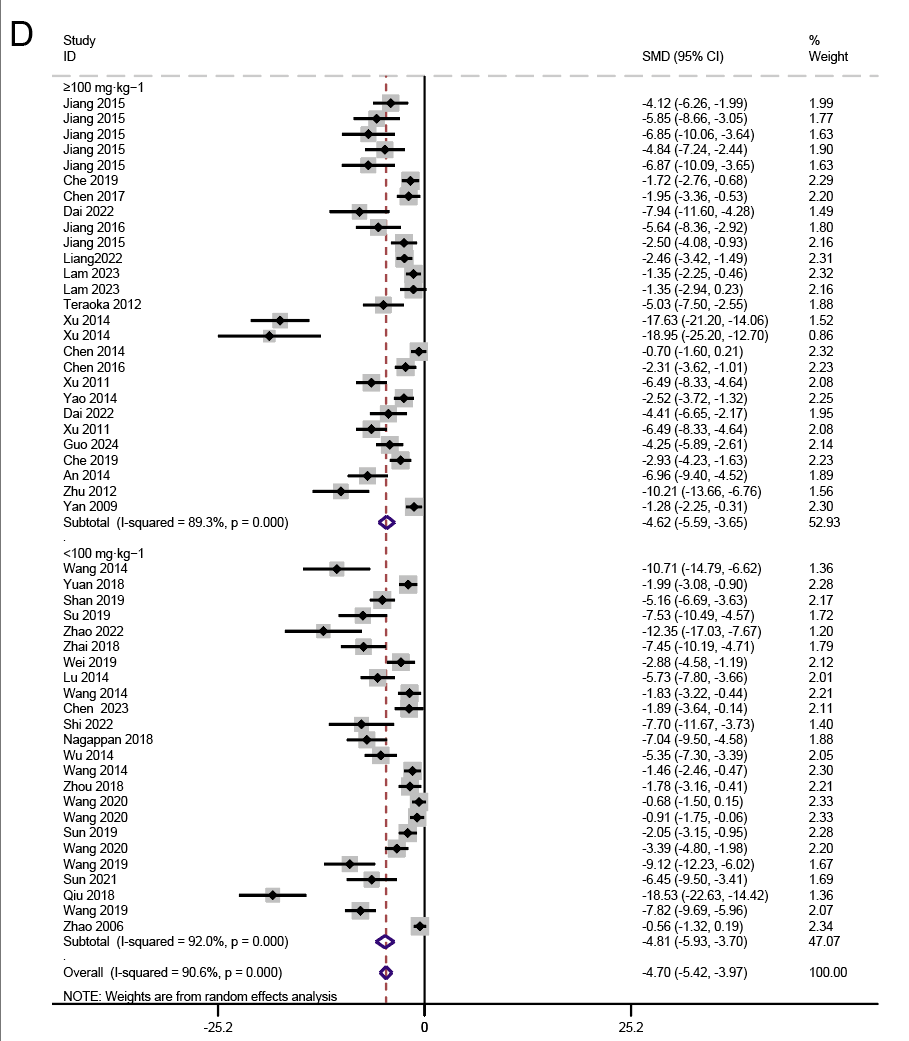


**Supplementary figure 1.** Sugroup analysis of pooled estimates of AST

(A) Therapeutic drugs (B) Animal species (C) Modeling drugs (D) Dosage


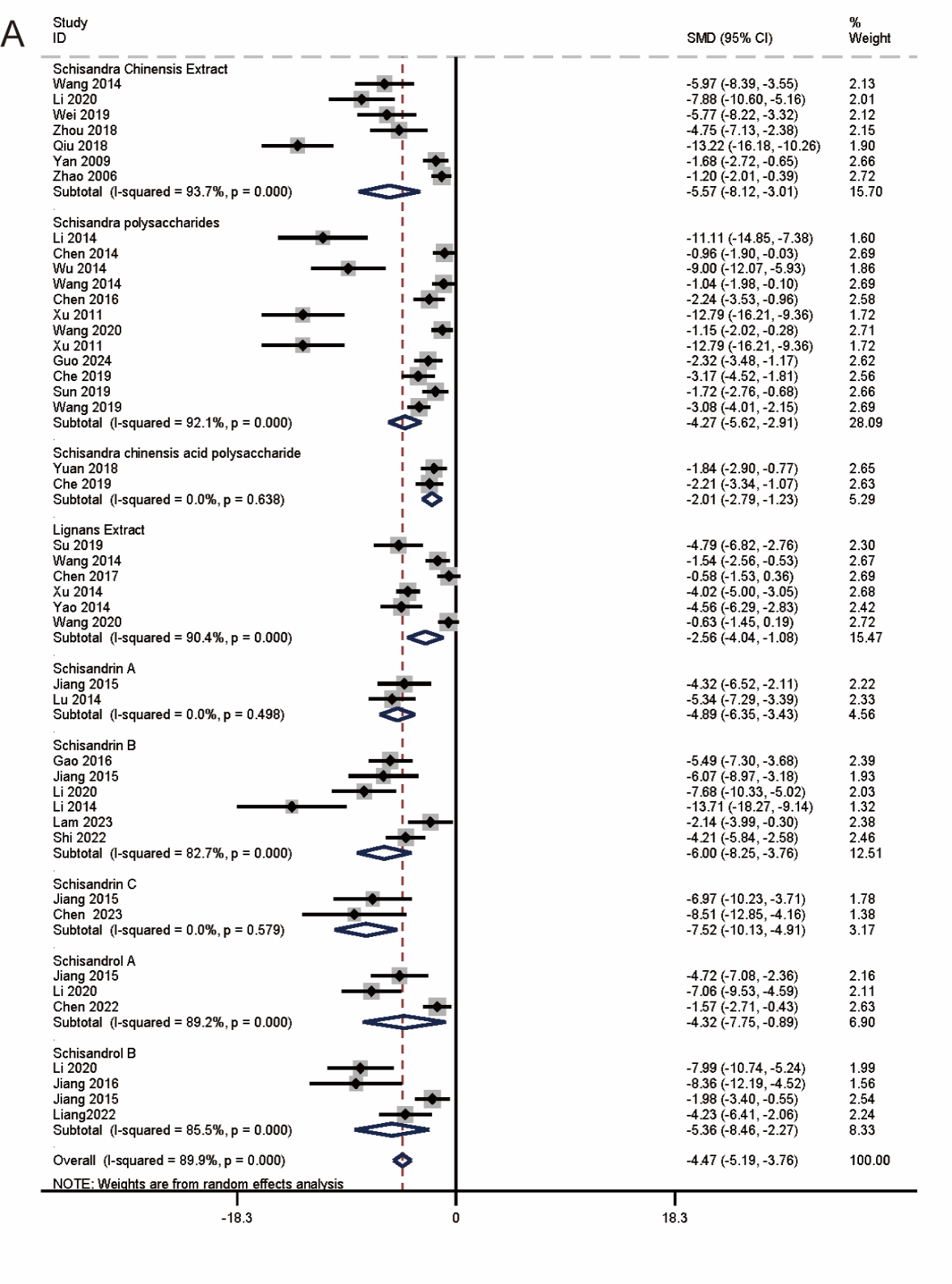


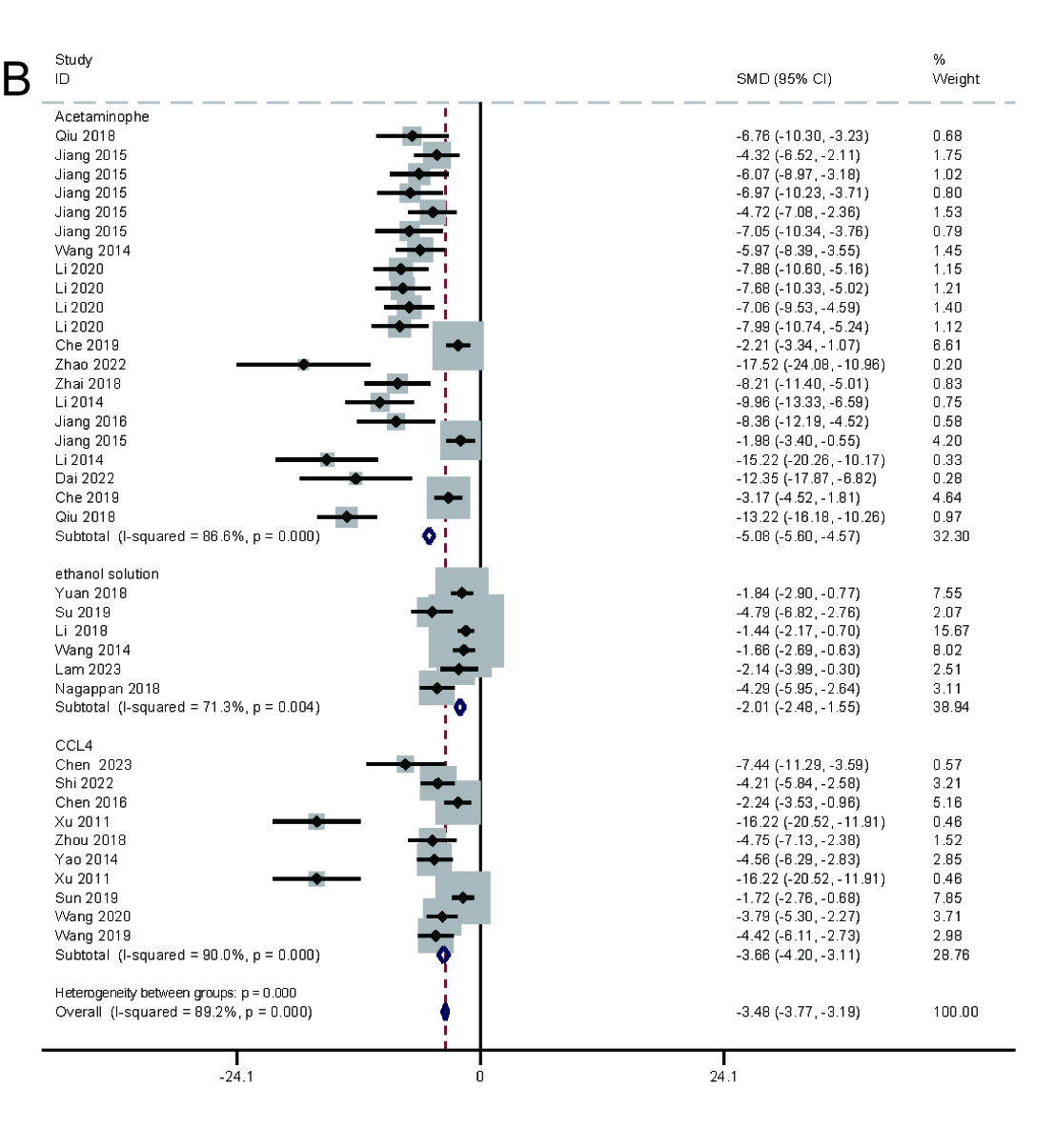


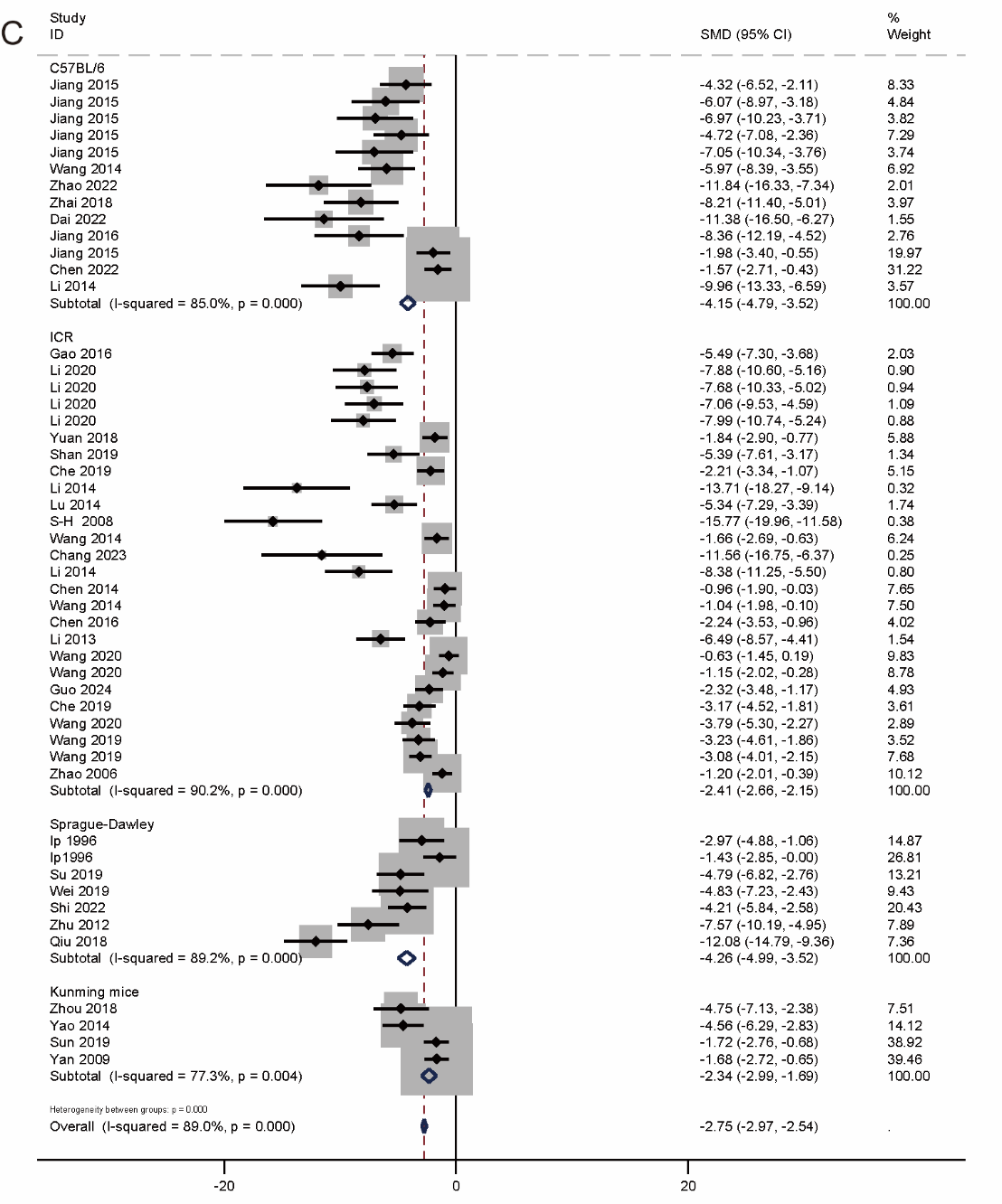


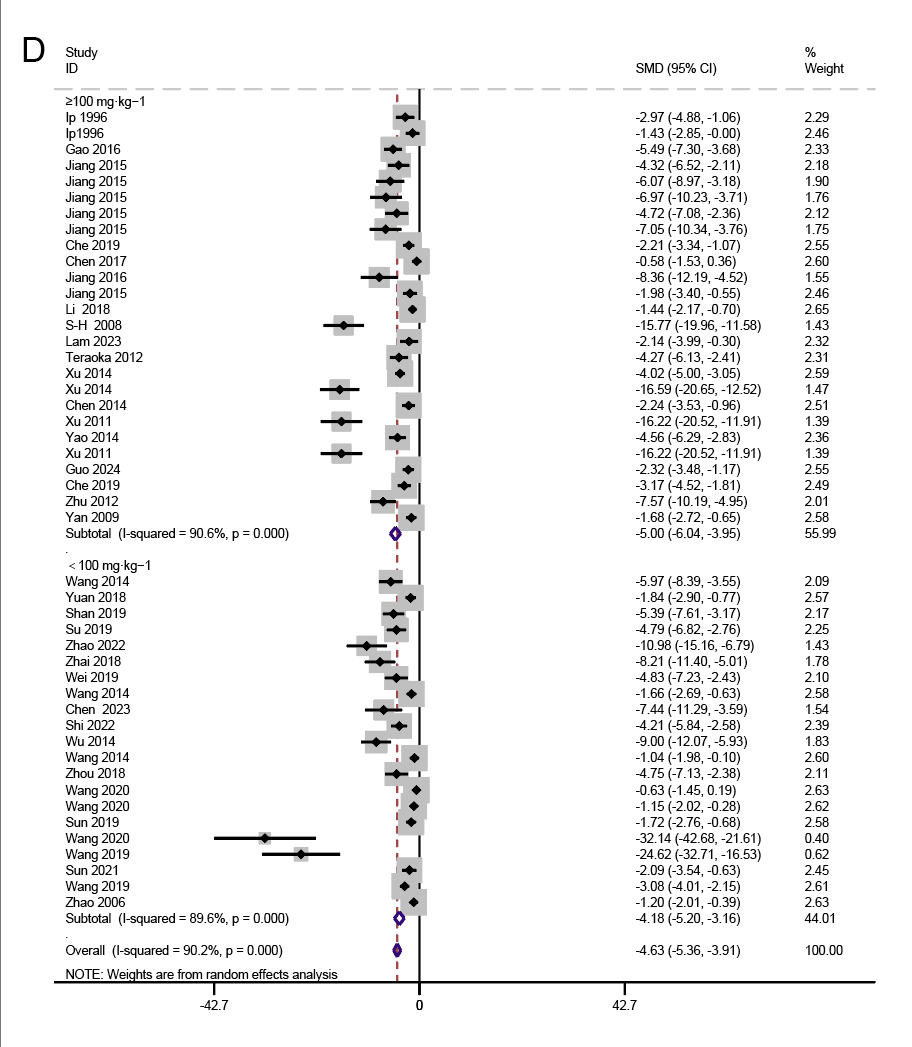


**Supplementary figure 2.** Subgroup analysis of pooled estimates of ALT

(A) Therapeutic drugs (B) Modeling drugs (C) Animal species (D) Dosage


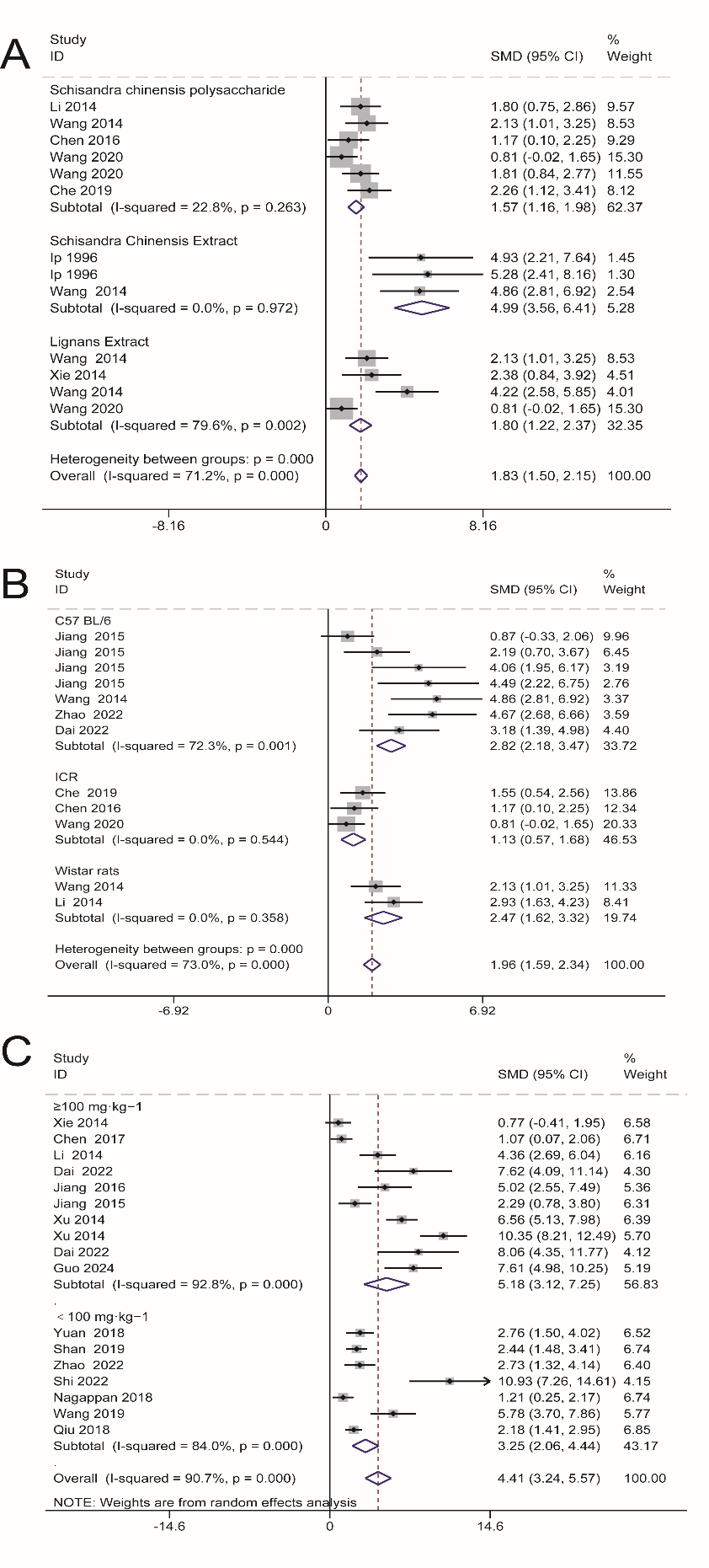


**Supplementary figure 3** Subgroup analysis of pooled estimates of SOD

(A) Therapeutic drugs (B) Animal species (C) Dosage


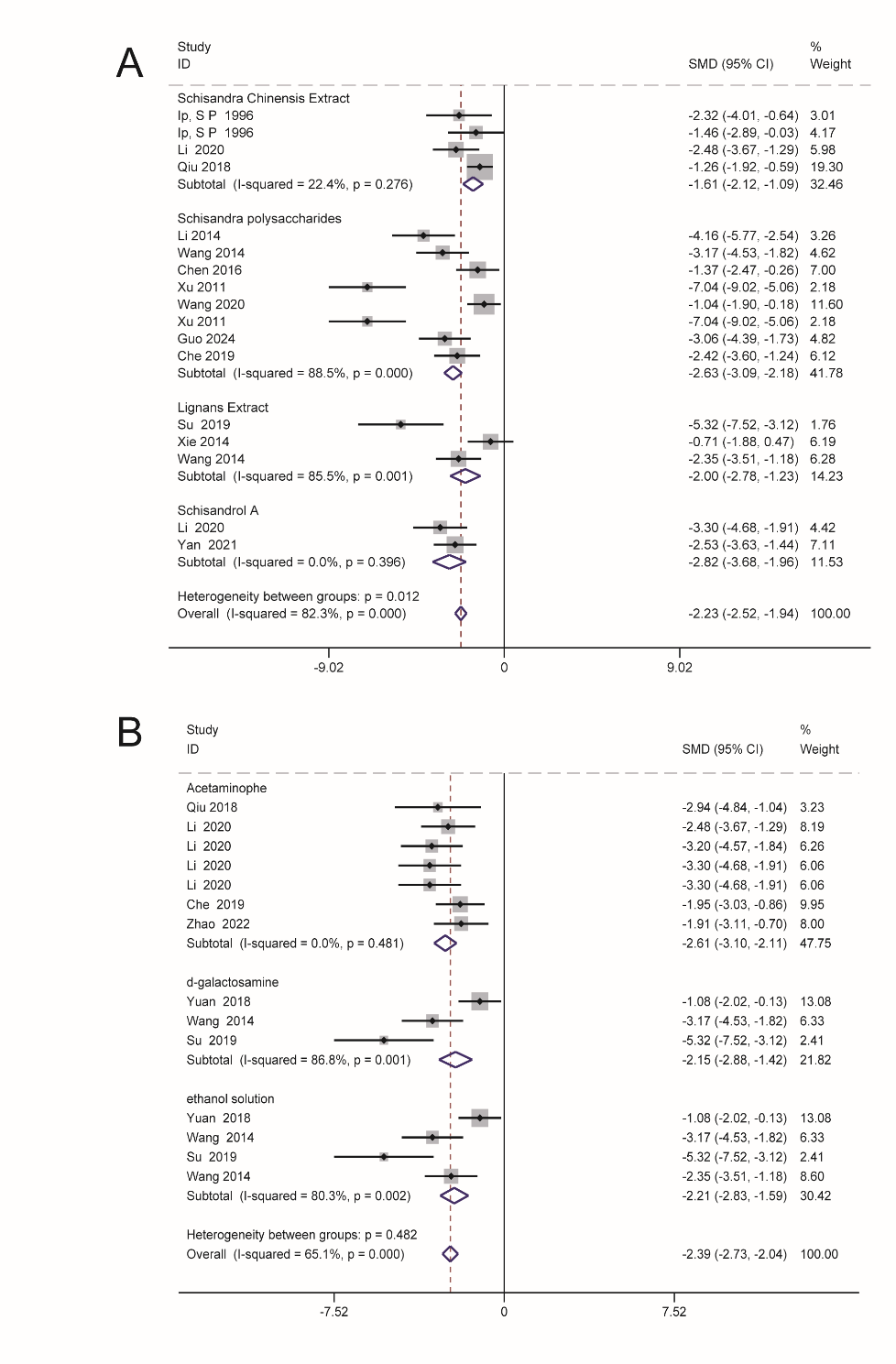

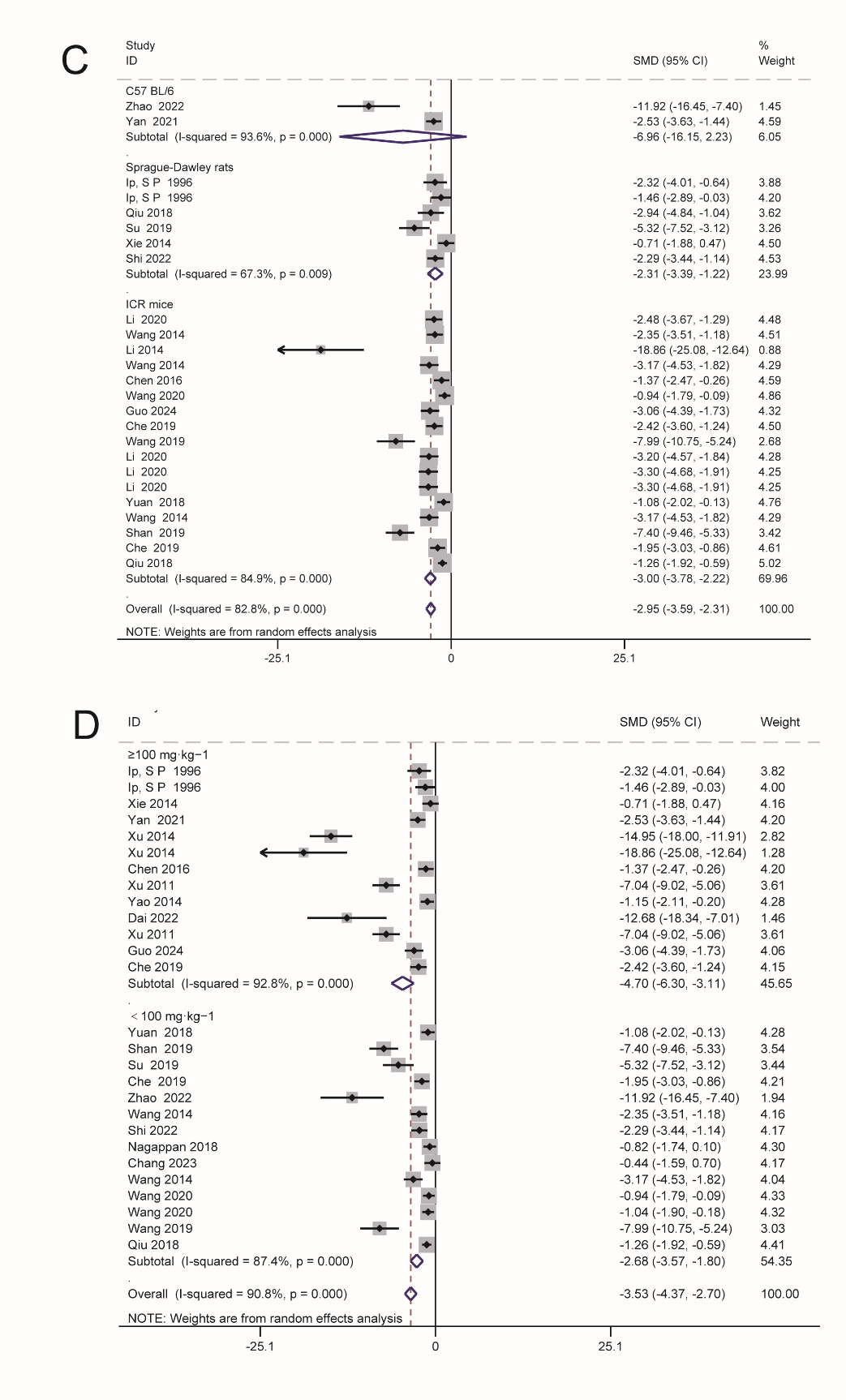


**Supplementary figure 4** Subgroup analysis of pooled estimates of MDA

(A) Therapeutic drugs (B) Modeling drugs (C) Animal species (D)Dosage


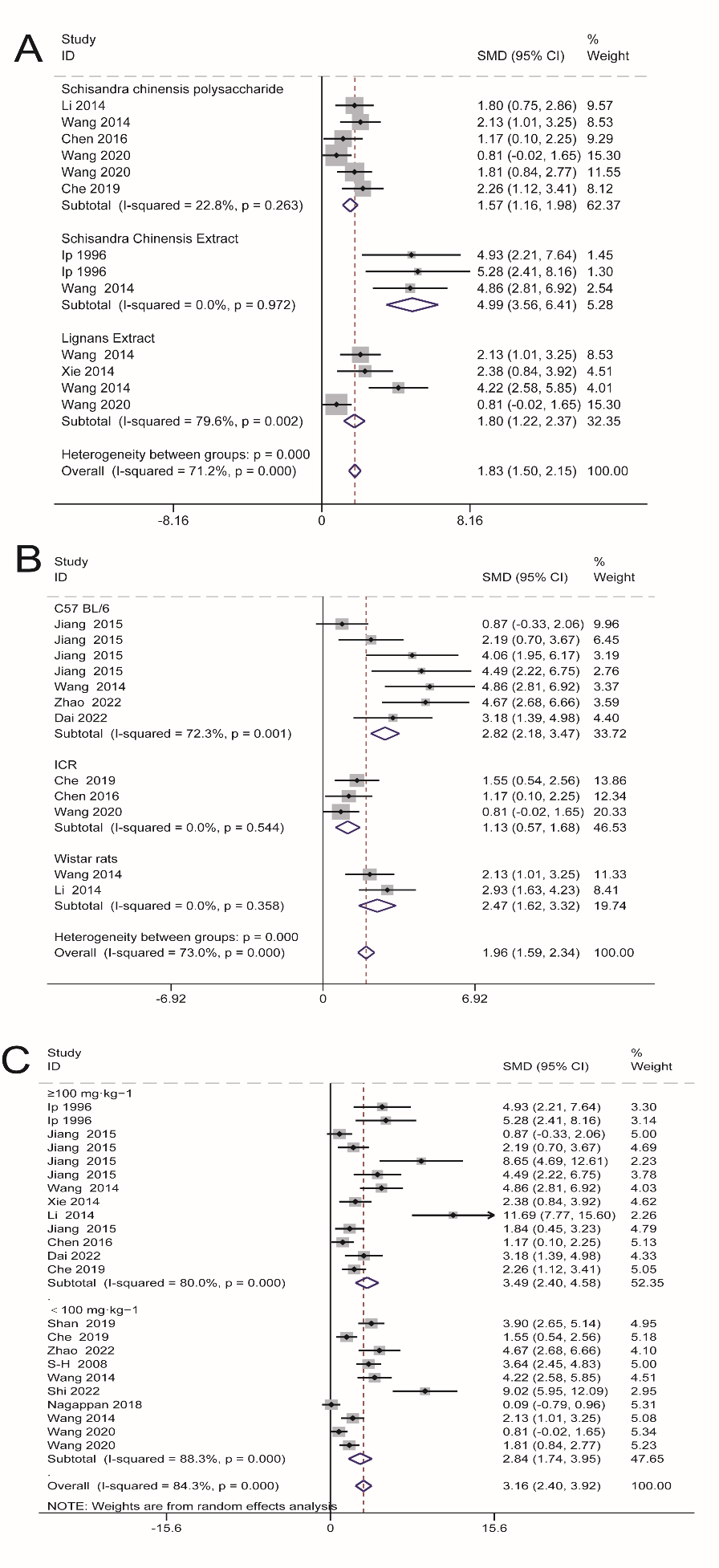


**Supplementary figure 5** Subgroup analysis of pooled estimates of GSH

(A) Therapeutic drugs (B) Animal species (C) Dosage


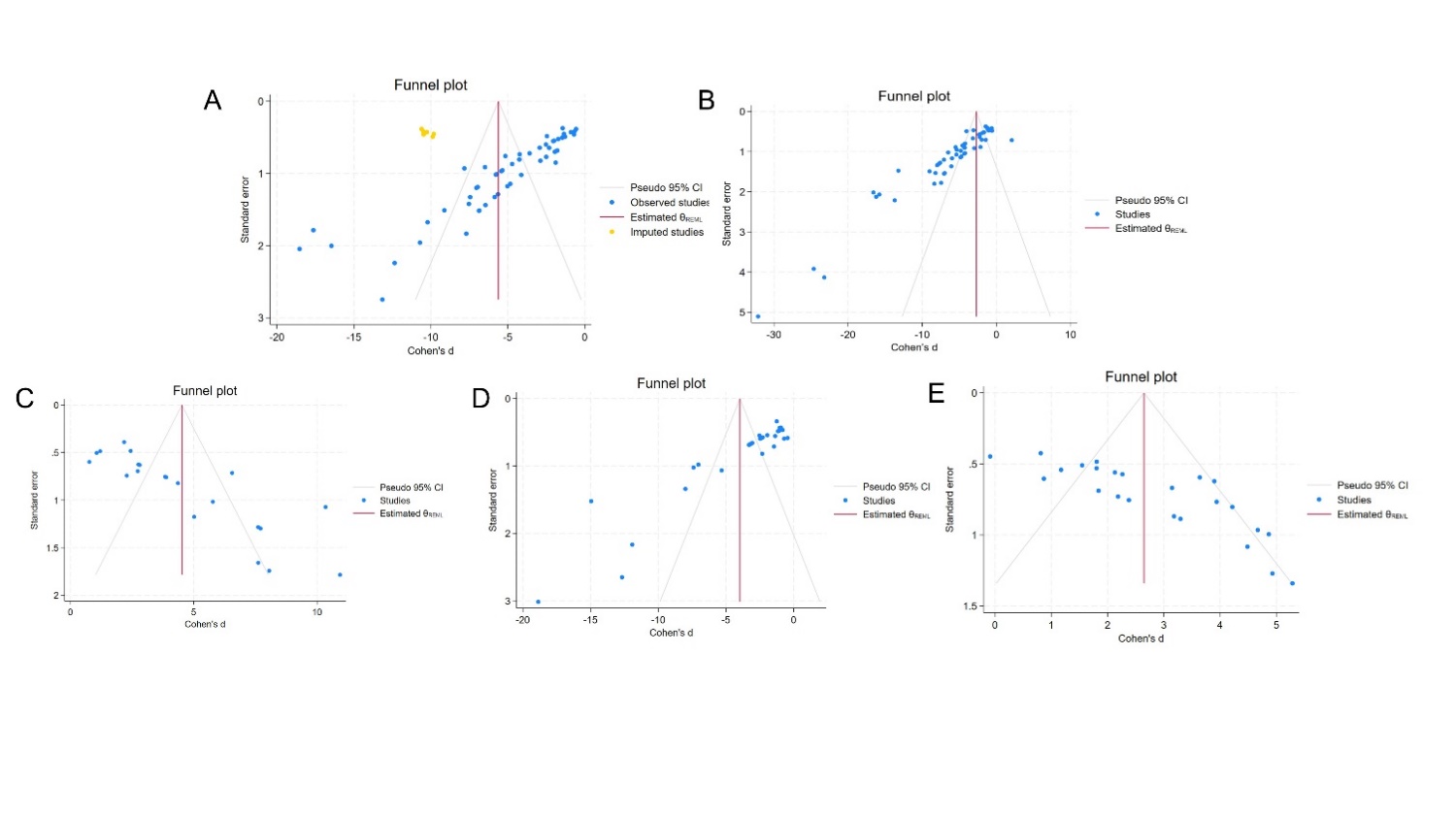


**Supplementary figure 6. Trim-and-fill analysis**

(A)AST; (B)ALT; (C)SOD; (D)MDA;(E) GSH
